# A novel transgenic mouse model of growth plate dysplasia reveals that decreased chondrocyte proliferation due to chronic ER stress is a key factor in reduced bone growth

**DOI:** 10.1242/dmm.013342

**Published:** 2013-09-12

**Authors:** Benedetta Gualeni, M. Helen Rajpar, Aaron Kellogg, Peter A. Bell, Peter Arvan, Raymond P. Boot-Handford, Michael D. Briggs

**Affiliations:** 1 Wellcome Trust Centre for Cell-Matrix Research, Faculty of Life Sciences, University of Manchester, Manchester, M13 9PT, UK; 2 University of Michigan Health System, Ann Arbor, MI 48109, USA

## Abstract

Disease mechanisms leading to different forms of chondrodysplasia include extracellular matrix (ECM) alterations and intracellular stress resulting in abnormal changes to chondrocyte proliferation and survival. Delineating the relative contribution of these two disease mechanisms is a major challenge in understanding disease pathophysiology in genetic skeletal diseases and a prerequisite for developing effective therapies. To determine the influence of intracellular stress and changes in chondrocyte phenotype to the development of chondrodysplasia, we targeted the expression of the G2320R mutant form of thyroglobulin to the endoplasmic reticulum (ER) of resting and proliferating chondrocytes. Previous studies on this mutant protein have shown that it induces intracellular aggregates and causes cell stress and death in the thyroid gland. The expression and retention of this exogenous mutant protein in resting and proliferating chondrocytes resulted in a chronic cell stress response, growth plate dysplasia and reduced bone growth, without inducing any alterations to the architecture and organization of the cartilage ECM. More significantly, the decreased bone growth seemed to be the direct result of reduced chondrocyte proliferation in the proliferative zone of growth plates in transgenic mice, without transcriptional activation of a classical unfolded protein response (UPR) or apoptosis. Overall, these data show that mutant protein retention in the ER of resting and proliferative zone chondrocytes is sufficient to cause disrupted bone growth. The specific disease pathways triggered by mutant protein retention do not necessarily involve a prototypic UPR, but all pathways impact upon chondrocyte proliferation in the cartilage growth plate.

## INTRODUCTION

The chondrodysplasias are a heterogeneous group of rare genetic diseases for which there are no effective therapies. Current research is therefore focused on understanding disease mechanisms and identifying potential therapeutic targets. The various chondrodysplasia phenotypes can arise from a broad spectrum of defects in either cartilage-specific structural proteins, metabolic processes or growth plate regulation that ultimately disturb endochondral ossification (Kornak and Mundlos, 2003; Warman et al., 2011). However, it has become increasingly evident that two interconnected pathways act synergistically to define the chondrodysplastic phenotype. On the one hand, disease-causing mutations disturb the complex extracellular matrix (ECM) network, altering the mechanical properties of the ECM and interfering with signalling pathways regulating endochondral ossification (Beier and LuValle, 2002; Chen and Deng, 2005; Cortes et al., 2009; Ishijima et al., 2012; Klüppel et al., 2005; Raducanu et al., 2009; Ruiz-Perez and Goodship, 2009; Wang et al., 2002; Yoon et al., 2005). On the other hand, intracellular stress is triggered in chondrocytes synthesising mutant proteins, causing alterations in the secretory pathway, disturbing normal cell metabolism and proliferation, and, in extreme cases, leading to cell death (Nundlall et al., 2010; Piróg-Garcia et al., 2007; Rajpar et al., 2009; Saito et al., 2009; Tsang et al., 2007).

Notwithstanding disease-specific features, phenotypically similar chondrodysplasias that are caused by different mutations can share some pathophysiological similarities. These can include the retention of mutant protein, co-retention of other interacting proteins, endoplasmic reticulum (ER) stress, reduced chondrocyte proliferation, increased and/or spatially dysregulated chondrocyte apoptosis, disturbed chondrocyte differentiation, and finally, altered signalling pathways (Cortes et al., 2009; Forlino et al., 2005; Gualeni et al., 2010; Nundlall et al., 2010; Piróg-Garcia et al., 2007; Posey et al., 2009; Raducanu et al., 2009; Rajpar et al., 2009; Rodgers et al., 2007; Sahni et al., 2001; Suleman et al., 2012; Wang et al., 2007; Wang et al., 2002). Therefore, delineating the relative contributions of intra- and extracellular disease mechanisms and evaluating the comparative influences of reduced chondrocyte proliferation and increased or dysregulated apoptosis on long bone growth are major challenges in understanding disease pathology in a broad range of genetic skeletal diseases, and is a prerequisite for identifying therapeutic targets.

**TRANSLATIONAL IMPACT**

**Clinical issue**

Chondrodysplasias are a clinically and genetically heterogeneous group of rare diseases for which there are no effective treatments. To identify potential therapeutic targets for these debilitating genetic skeletal disorders, current research is focused on understanding the underlying pathogenic mechanisms. Disease mechanisms leading to different forms of chondrodysplasia include extracellular matrix (ECM) alterations and intracellular stress leading to abnormal changes in chondrocyte proliferation and survival. Delineating the relative contribution of these interconnected mechanisms to disease onset is a major challenge. Targeted transgenic mouse models of specific chondrodysplasias have established that mutant protein expression causes chronic endoplasmic reticulum (ER) stress, reduced chondrocyte proliferation, increased and/or spatially dysregulated apoptosis, and abnormal changes to the organization and architecture of the ECM. The complex pathology of these diseases cannot be readily dissected in these targeted mouse models, highlighting the need to develop novel transgenic mice that can be used to assess the distinct effects of intracellular stress and ECM alterations on chondrocytes and growth plate pathology.

**Results**

The authors targeted the expression of a mutant version of thyroglobulin, a protein known to accumulate in the ER and trigger intracellular stress in the thyroid gland, to chondrocytes in mice. The expression and retention of this exogenous mutant protein in resting and proliferating chondrocytes resulted in a chronic cell stress response, growth plate dysplasia and reduced bone growth, without inducing any alterations to the architecture and organization of the cartilage ECM. Interestingly, the decreased bone growth was the direct result of reduced chondrocyte proliferation in the proliferative zone of transgenic mouse growth plates and did not involve transcriptional activation of a classical unfolded protein response (UPR) or apoptosis.

**Implications and future directions**

This work provides a novel transgenic model for investigating the role of intracellular stress in chondrodysplasia development. Future studies using this transgenic model will identify new therapeutic targets and provide novel diagnostic and prognostic biomarkers for these diseases. Moreover, this study shows for the first time that mutant protein retention in the ER of chondrocytes is sufficient to cause disrupted bone growth by reducing chondrocyte proliferation. Intriguingly, the specific disease pathways triggered by mutant protein retention do not necessarily involve a prototypic UPR. This finding compels a paradigm shift in our understanding of ER stress in the context of genetic diseases. Understanding why and how different misfolded mutant proteins evoke UPR-independent stress pathways is an exciting new area in human pathobiology.

Targeted transgenic mice, such as those modelling pseudoachondroplasia (PSACH) and multiple epiphyseal dysplasia (MED) caused by antimorphic mutations in cartilage oligomeric matrix protein (COMP: p.D469del and p.T585M; PSACH) and matrilin-3 (p.V194D; MED), have established that mutant protein retention causes chronic ER stress, reduced chondrocyte proliferation, increased and/or spatially dysregulated apoptosis, and abnormal changes to the organisation and architecture of the ECM (Bell et al., 2013; Leighton et al., 2007; Nundlall et al., 2010; Piróg-Garcia et al., 2007; Suleman et al., 2012). However, the complex pathology of PSACH and MED cannot readily be dissected in these mouse models and only through the use of novel transgenic mouse approaches can we begin to delineate the relative contributions of these interconnected disease pathways.

In this context, the aim of this study was to dissect the relative contribution of intracellular stress in resting and proliferating chondrocytes to the development of chondrodysplasia by targeting the expression of an exogenous mutant protein, known to accumulate in the ER and trigger intracellular stress, predominantly in these cell types. This approach was achieved by expressing the G2320R mutant form of thyroglobulin (Tg) under the collagen type II promoter to generate a novel mouse model. A similar approach has been used previously to generate a mouse model expressing the L2263P mutant form of Tg (Rajpar et al., 2009), known to induce ER stress (Kim et al., 1996), exclusively in hypertrophic chondrocytes. This mouse model confirmed the central role of hypertrophic chondrocyte ER stress in the pathogenesis of metaphyseal dysplasia type Schmid (MCDS). However, owing to the restricted expression of type X collagen to hypertrophic chondrocytes, both this novel phenocopy and also targeted mouse models of MCDS are not able to address the fundamental role of chronic ER stress in chondrocytes within the resting and proliferative zone, which are the essential precursors for hypertrophy.

Tg is a 660-kDa homodimeric protein produced by the thyroid gland. It is the major secretory glycoprotein produced by thyrocytes and is the precursor protein for thyroid hormone synthesis (Malthièry et al., 1989). During synthesis in the ER, Tg monomers assemble to form large multimers associated with ER-resident molecular chaperones. Monomers released from these complexes can then be glycosylated and subsequently dimerize to form the mature protein (Lee et al., 2009), with the cholinesterase-like (ChEL) domain at the C-terminus of the protein being important for Tg dimerization (Lee et al., 2008). The spontaneous G2320R missense mutation in the ChEL domain of Tg is responsible for congenital hypothyroidism in *rdw/rdw* rats (Kim et al., 2000) through a mechanism that involves protein retention in the ER and ablated synthesis of thyroid hormones. The Tg-G2320R protein (Tg^rdw^) that accumulates in the ER is misfolded with abnormal and persistent exposure of free cysteine thiols, which results in the strong binding of ER-resident oxidoreductases [in particular ERp72, but also protein disulfide-isomerase (PDI) and ERp57]. The association of Tg^rdw^ with ERp72 is believed to render a subfraction of the mutant Tg resistant to ER-associated degradation (ERAD) and leads to cell death, suggesting a possible gain of toxic function (antimorphic) for the mutant protein in thyrocytes (Menon et al., 2007).

In this study we generated transgenic mice expressing Tg^rdw^ under the collagen type II promoter (*Col2-Tg*^rdw^ mice), thereby causing intracellular stress predominantly in resting and proliferating chondrocytes, and observed that transgenic mice develop a severe chondrodysplasia without any detectable alterations to ECM composition or increased levels of chondrocyte apoptosis. We show that intracellular stress alone, in the absence of transcriptional activation of the unfolded protein response (UPR), is sufficient to cause chondrodysplasia through reduced chondrocyte proliferation.

## RESULTS

### Generation of transgenic mice expressing Tg^rdw^ under the *Col2* promoter

**Fig. 1.**
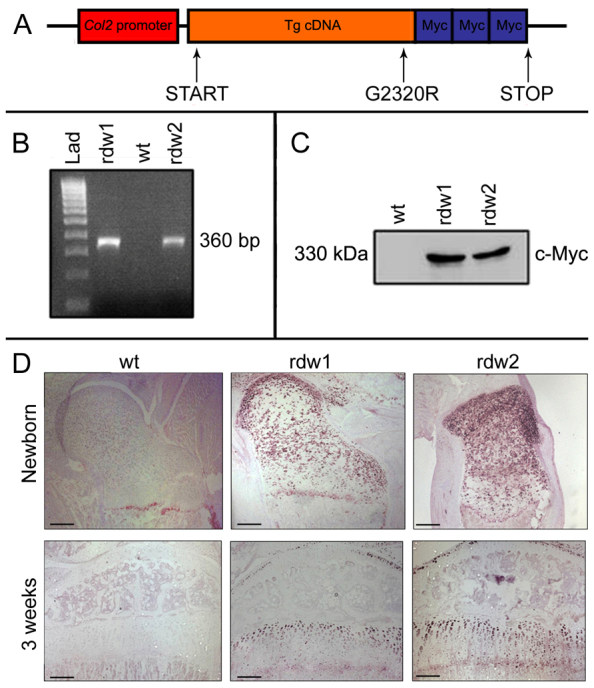
**Generation of the *Col2-Tg*^rdw^ transgenic mouse model.** (A) Schematic of the transgene showing how the expression of G2320R (rdw) thyroglobulin (Tg) was driven by the type II collagen (*Col2*) promoter (*Col2-Tg*^rdw^). Three Myc tags (Myc) were included at the C-terminus of the transgenic protein to allow detection. (B) Agarose gel electrophoresis of PCR-amplified mouse genomic DNA. A distinct band at ∼360 bp was observed in both *Col2-Tg*^rdw^ transgenic animals (rdw1 and rdw2), whereas no DNA product was amplified from wild-type animals (wt). Hyperladder IV was used as molecular weight marker (Lad). (C) Western blot analysis demonstrating that a band at ∼330 kDa, consistent with the size of Tg^rdw^ (including tags), was detected in *Col2-Tg*^rdw^ transgenic mice (rdw1 and rdw2) using an anti-Myc antibody (c-Myc). Comparable levels of transgene protein were detected in the two different *Col2-Tg*^rdw^ lines, whereas no transgene expression was observed in wild-type animals (wt). (D) Immunohistochemistry using an anti-Myc antibody demonstrated that no transgenic protein was detected in the growth plate cartilage of newborn or 3-week-old wild-type mice (wt), whereas mutant Tg^rdw^ transgenic protein was clearly retained within chondrocytes of growth plate and articular cartilages of the two transgenic lines (rdw1 and rdw2) at birth and at 3 weeks of age. There was no evidence of mutant Tg^rdw^ transgenic protein secretion in the cartilage ECM of either mouse line. Scale bars: 100 μm.

The expression of Tg^rdw^ was targeted to mouse chondrocytes by the type II collagen promoter (*Col2-Tg*^rdw^) and, for easier detection of the mutant protein, three Myc tags were included at the C-terminus of the transgenic protein (Fig. 1A; supplementary material Fig. S1). Transgenic mice were generated by pronuclear injection and two independent lines that had incorporated the transgene (Fig. 1B) and expressed Tg^rdw^ in chondrocytes (Fig. 1C) were analyzed separately in the first instance. We also confirmed that Tg^rdw^ protein expression was localized to type-II-collagen-expressing chondrocytes found in the growth plate, costal and articular cartilages (Fig. 1D; supplementary material Fig. S1). The intracellular accumulation of mutant Tg^rdw^ protein in the chondrocytes of newborn *Col2-Tg*^rdw^ mice is consistent with targeted mouse models of PSACH, MED and MCDS, and confirms that mutant protein retention (i.e. Tg^rdw^, COMP, matrilin-3 and type X collagen) precedes the onset of clinical symptoms such as reduced bone growth (supplementary material Table S1).

By real-time qPCR on genomic DNA, we observed that a larger transgene copy number was present in one mouse line compared with the other (our unpublished observations), but all subsequent analyses showed no discernible differences in the morphological and biochemical phenotype of the two lines, and therefore data obtained from the two lines were pooled where appropriate for the final analyses. Measurements obtained from male and female mice followed similar trends and the data presented herein are from the males.

### The expression of Tg^rdw^ in chondrocytes causes short-limb dwarfism in transgenic mice

Transgenic mice expressing *Col2-Tg*^rdw^ were viable and were either normal or slightly smaller than wild-type mice at birth. This finding is consistent with our previously published targeted mouse models of chondrodysplasia (supplementary material Table S1) and is a well-documented clinical feature in individuals with PSACH, MED and MCDS (Briggs and Chapman, 2002; Lachman et al., 1988; Leighton et al., 2007; Piróg-Garcia et al., 2007; Rajpar et al., 2009; Suleman et al., 2012).

**Fig. 2.**
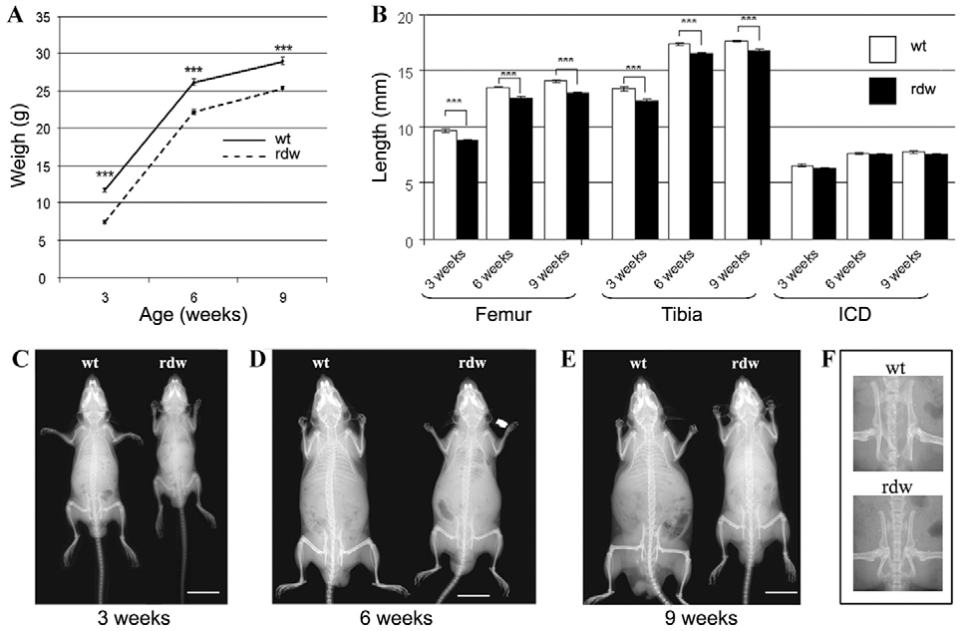
***Col2-Tg*^rdw^ transgenic mice develop a short-limb dwarfism.** (A) The weights of wild-type (wt; solid line) and *Col2-Tg*^rdw^ transgenic (rdw; dashed line) mice were collected at 3, 6 and 9 weeks of age. *Col2-Tg*^rdw^ animals were significantly lighter than age-matched controls at all the age points analyzed (***P<0.0001). (B) Bone length measurements of the femur, tibia and inner canthal distance (ICD) of wild-type (wt; white columns) and *Col2-Tg*^rdw^ (rdw; black columns) animals. The long bones of transgenic mice were significantly shorter than normal at all the age points examined (***P<0.0001), whereas ICD was not affected by the expression of the *Col2-Tg*^rdw^ transgene. (C–E) Bone measurements were performed on radiographies of wild-type (wt) and transgenic (rdw) mice at 3 (C), 6 (D) and 9 (E) weeks of age. Scale bars: 15 mm. (F) Radiographs of the pelvis of 3-week-old wild-type (wt) and transgenic (rdw) mice, demonstrating the hip dysplasia that *Col2-Tg*^rdw^ animals occasionally developed.

By 3 weeks of age, *Col2-Tg*^rdw^ mice were significantly lighter than their wild-type equivalents (Fig. 2A). For example, at 3 weeks of age transgenic mice were 35.5% lighter (*P*<0.0001), at 6 weeks they were 16.6% lighter (*P*<0.0001) and finally by 9 weeks they were 14.9% lighter (*P*<0.0001) than age-matched wild-type mice (*n*≥19 mice per genotype).

At 3 weeks of age the long bones of *Col2-Tg*^rdw^ mice were shorter than those of age-matched wild-type controls, signifying a defect in endochondral bone growth (Fig. 2B). Indeed, the femurs and tibiae of *Col2-Tg*^rdw^ mice were significantly shorter than age-matched controls (Fig. 2B–E; *P*<0.0001) at all time points examined (3 weeks: 8.8% and 8.1%, 6 weeks: 6.4% and 4.6%, and 9 weeks: 7.7% and 4.9%, respectively; *n*≥30 mice per genotype). However, the inner canthal distance (ICD) in transgenic animals was the same as controls at all ages analyzed (*n*≥16 mice per genotype), confirming that the expression of the transgene affected endochondral and not intramembranous bone formation (Fig. 2B).

A mild hip dysplasia characterized by an increase in the angle of deflection from the vertical of the tuberosity of the ischium was detected in ∼4% of *Col2-Tg*^rdw^ mice from 3 weeks of age (Fig. 2F). This feature is often reported in other targeted mouse models of chondrodysplasias, including those of PSACH (Piróg-Garcia et al., 2007; Suleman et al., 2012).

### *Col2-Tg*^rdw^ mouse growth plates are dysplastic with disorganized chondrocyte columns and distinct areas of hypocellularity, but with no major alteration to the cartilage ECM

**Fig. 3.**
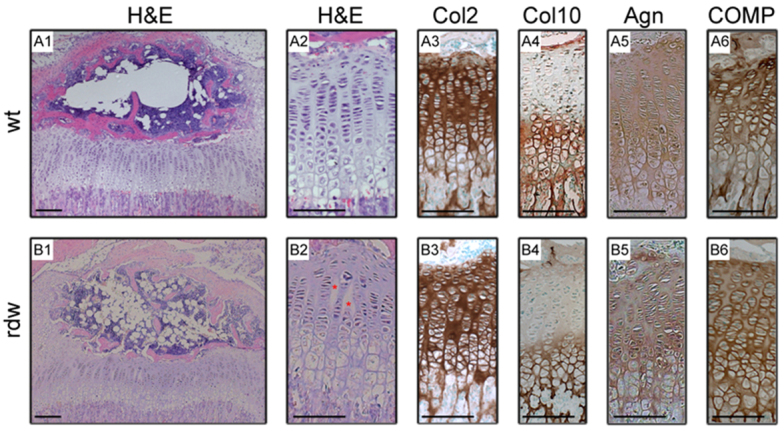
**The overall architecture and ECM composition in the growth plate of transgenic mice is normal.** The extension and organization of the growth plate was similar in wild-type (wt) and transgenic (rdw) animals (A1 and B1, respectively; close ups in A2 and B2), even though more areas of hypocellularity were detected in the growth plates of *Col2-Tg*^rdw^ mice (see red asterisks in B2). The relative levels and distribution of important ECM structural molecules such as type II collagen (Col2; A3 and B3), type X collagen (Col10; A4 and B4), aggrecan (Agn; A5 and B5) and cartilage oligomeric matrix protein (COMP; A6 and B6) in the growth plates of *Col2-Tg*^rdw^ mice were comparable to those in wild-type animals. Scale bars: 100 μm.

The overall development of the growth plate and formation of the secondary centre of ossification was relatively normal in *Col2-Tg*^rdw^ transgenic mice up to 3 weeks of age (Fig. 3; supplementary material Fig. S2). The different zones of the growth plate (resting, proliferating and hypertrophic) were clearly distinguishable (Fig. 3A1,B1; with higher magnification shown in A2 and B2), although some abnormal areas of hypocellularity were apparent in the growth plates of transgenic mice (Fig. 3B2; red asterisks). Moreover, in some, but not all, *Col2-Tg*^rdw^ growth plates the chondrocyte columns in the proliferative zone were grossly disorganized and this pathological feature presented as a continuum from mild to severe (supplementary material Fig. S3).

Morphometric analysis confirmed that there were no differences in the overall width of the growth plate (wild type 246±15 μm; *Col2-Tg*^rdw^ 209±12 μm) or the relative proportions of the resting (wild type 11±1%; *Col2-Tg*^rdw^ 13±1%), proliferative (wild type 52±2%; *Col2-Tg*^rdw^ 49±1%) and hypertrophic (wild type 37±1%; *Col2-Tg*^rdw^ 38±1%) zones. Furthermore, the number of hypertrophic chondrocytes in individual cell columns within the hypertrophic zone did not differ between genotypes (wild type 6.76±0.4; *Col2-Tg*^rdw^ 6.52±0.17) (supplementary material Fig. S4; three sections per mouse and three mice per genotype were analyzed).

**Fig. 4.**
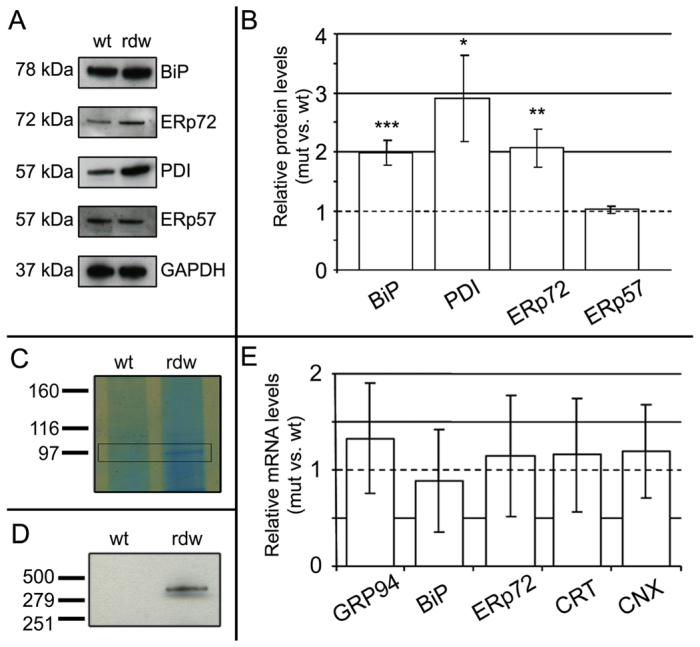
**Tg^rdw^ aggregates with ER-resident oxidoreductases and PDIs, but no transcriptional UPR is triggered in the chondrocytes of transgenic mice.** (A) Representative western blots of the total protein extracted from chondrocytes of 5-day-old wild-type (wt) and *Col2-Tg*^rdw^ (rdw) mice analyzed for BiP, ERp72, PDI, ERp57 and GAPDH as a loading control. (B) Densitometry analysis was performed on western blots of three different biological replicates and showed significantly increased levels of BiP, PDI and ERp72 in *Col2-Tg*^rdw^ chondrocytes compared with wild type (*P<0.01; **P<0.001; ***P<0.0001), whereas no difference was detected in ERp57. (C) Proteins extracted from 5-day-old wild-type (wt) and *Col2-Tg*^rdw^ (rdw) mouse chondrocytes were separated on a 4–12% SDS-PAGE gel and stained with Instant*Blue* Coomassie to visualize the full protein profile of the two different genotypes. The only major detectable difference was a band at ∼97 kDa that was present in *Col2-Tg*^rdw^ mice and absent in controls (black frame). After excision and analysis by mass spectrometry, the protein was found to correspond to GRP94. (D) Tg^rdw^ was immunoprecipitated using an anti-Myc antibody conjugated with agarose beads. The expected precipitation of the transgenic protein was verified by western blotting before proceeding with mass spectrometry to identify co-precipitating proteins. A correctly sized band at 330 kDa was detected in *Col2-Tg*^rdw^ mice (rdw), whereas no band was detected in controls (wt). (E) Real-time qPCR showed that the relative mRNA levels of GRP94, BiP, ERp57 (PDIA3), calreticulin (CRT) and calnexin (CNX) transcripts were normal in the chondrocytes of *Col2-Tg*^rdw^ mice.

Importantly, mutant Tg^rdw^ protein retention did not cause significant co-retention of key cartilage ECM proteins. For example, type II collagen (Fig. 3A3,B3), type X collagen (Fig. 3A4,B4), aggrecan (Fig. 3A5,B5) and COMP (Fig. 3A6,B6) were secreted and normally distributed in the cartilage ECM of transgenic mice. In addition, mass spectrometry (MS)-based proteomics was also performed on sequential cartilage extractions to study the differential extractability of structural ECM components from the cartilage of wild-type and transgenic animals. This analysis confirmed that the retention of mutant Tg^rdw^ protein did not cause alterations to the extractability of 29 structural ECM components from the cartilage of 3-week-old mice (supplementary material Table S2), thereby confirming that the organization and integrity of the cartilage ECM was not significantly affected in *Col2-Tg*^rdw^ mice. The only significant differences were: α1(IX) collagen and osteomodulin, which showed increased extractability from *Col2-Tg*^rdw^ cartilage in buffer 1; α1(II) collagen, which was less extractable from *Col2-Tg*^rdw^ cartilage in buffer 1; α3(VI) collagen and fibronectin, which were both more easily extracted from *Col2-Tg*^rdw^ cartilage in buffer 2. This disease profile contrasts sharply with targeted mouse models of PSACH-MED in which there are highly significant differences in the extractability of numerous cartilage structural proteins including various collagens, small leucine-rich proteoglycans and tenascins (supplementary material Table S2) (Bell et al., 2013).

The proteomic observations were supported in part by microarray analysis performed on the mRNA isolated from chondrocytes extracted from the cartilage of 5-day-old mice. This study did not show any major changes in the relative expression of 30 genes encoding cartilage structural proteins, with the exception of a slight but statistically significant downregulation in the expression of matrilin 1 (*Matn1*, −1.51-fold) and type X collagen (*Col10a1*, −1.61-fold) in *Col2-Tg*^rdw^ mice (supplementary material Table S3). However, the comparison of these data sets is limited because total protein quantity and their relative extractability will not necessarily be representative of gene expression levels.

### A transcriptional UPR is not induced in the chondrocytes of *Col2-Tg*^rdw^ transgenic mice

We hypothesized that ER accumulation of the mutant transgenic protein and the resulting ER stress might trigger the UPR or an oxidative stress response in the chondrocytes of *Col2-Tg*^rdw^ transgenic mice, similar to that observed in thyrocytes (Menon et al., 2007). Western blot analysis of total proteins extracted from the chondrocytes of 5-day-old mice showed increased levels of BiP (∼twofold, *P*<0.0001), PDI (∼threefold, *P*<0.01) and ERp72 (PDIA4; ∼twofold, *P*<0.001), but normal levels of ERp57 (PDIA3) in *Col2-Tg*^rdw^ mice (Fig. 4A,B); increased levels of GRP94 in *Col2-Tg*^rdw^ chondrocytes was confirmed by MS (Fig. 4C). We also analyzed the cartilage proteome generated by liquid-chromatography–tandem-mass-spectrometry (LC-MS/MS), which allowed us to determine the relative levels of an additional 20 chaperone proteins from 3-week-old mouse cartilage (Table 1). This analysis only identified significant differences in the levels of calreticulin (Calr: wild type: 4; *Col2-Tg*^rdw^: 8; *P*<0.05) and peroxiredoxin 1 (Prdx1: wild type: 4; *Col2-Tg*^rdw^: 9; *P*<0.03). These global findings were in contrast to candidate SDS-PAGE and western blotting (Fig. 4B), which showed moderate but significant increases in several chaperones and PDIs; these discrepancies might be due to the differences in the age of the mice used between the two studies (5 days versus 3 weeks).

**Table 1.**
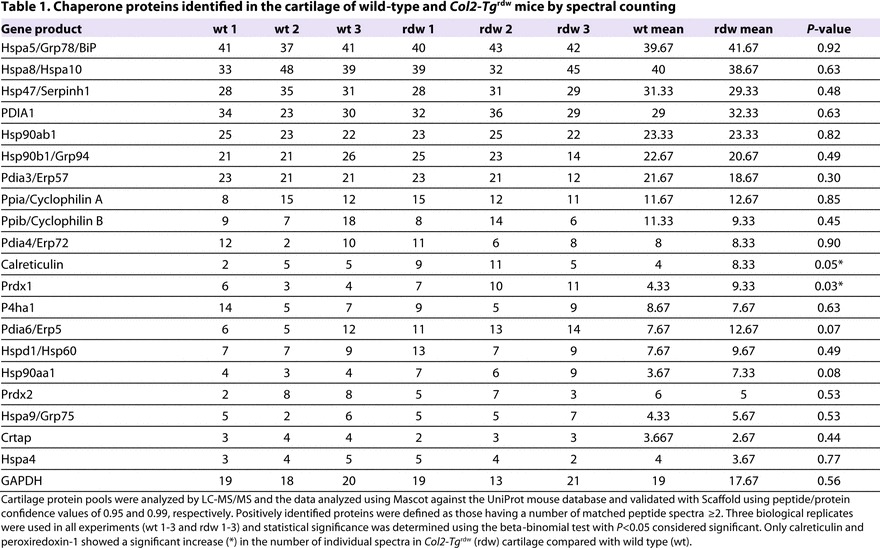
Chaperone proteins identified in the cartilage of wild-type and *Col2-Tg*^rdw^ mice by spectral counting

To identify the range of proteins (such as chaperones and foldases) that either interact directly with Tg^rdw^ or are present in ‘folding complexes’, we performed immunoprecipitation on the intracellular protein pool extracted from the chondrocytes of 5-day-old *Col2-Tg*^rdw^ mice. A subfraction of the immunoprecipitate was used to verify the expected presence of Tg^rdw^ (Fig. 4D), whereas the remainder was analyzed by LC-MS/MS to identify co-precipitating proteins. Tg^rdw^ interacted primarily with PDIA6, BiP and a number of ribosomal proteins (Table 2). Surprisingly, in this pull-down assay we were not able to detect an interaction between Tg^rdw^ and other members of the PDI protein family such as PDIA4, even though the relative levels were increased in *Col2-Tg*^rdw^ mutant chondrocytes (see Fig. 4A,B).

**Table 2.**
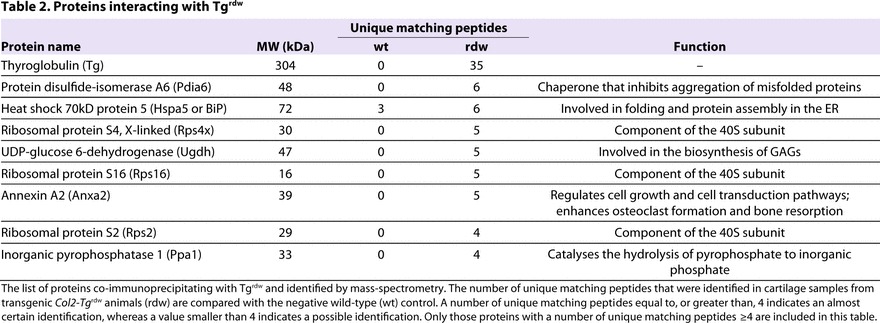
Proteins interacting with Tg^rdw^

We next performed real-time qPCR and microarray analysis on mRNA extracted from the chondrocytes of 5-day-old mice to determine the transcriptional consequences of expressing Tg^rdw^. This time point was specifically chosen because we and others have previously shown that mutant protein retention at birth elicits a robust UPR in mouse models of MED and MCDS by 5 days of age (Cameron et al., 2011; Nundlall et al., 2010; Rajpar et al., 2009). Real-time qPCR showed no relative increase in the mRNA levels of BiP, CRT, ERp72 (PDIA4) or GRP94 in *Col2-Tg*^rdw^ compared with wild-type mice (Fig. 4E). This finding was also confirmed by microarray analysis, which demonstrated that the mRNA levels of more than 150 genes associated with ER stress, ERAD and proteasomal degradation were normal in *Col2-Tg*^rdw^ mice, although slight (but statistically significant) changes were observed for a few genes, including: activation transcription factor 3 (Atf3, −1.26-fold), insulin-like growth factor 1 (Igf1, +1.92-fold), dolichol-phosphate (beta-D) mannosyltransferase 2 (Dpm2, +1.31-fold), proteasome (prosome, macropain) subunit, alpha type 4 (Psma4, +1.18-fold), proteasome (prosome, macropain) subunit, alpha type 7 (Psma7, +1.42-fold), DnaJ (Hsp40) homolog, subfamily C, member 9 (Dnajc9, +1.39-fold), DnaJ (Hsp40) homolog, subfamily C, member 15 (Dnajc15, +1.32-fold) and heat shock protein c171 (Hspc171, +1.42-fold) (supplementary material Table S3).

Overall, these data provide evidence of a moderate cellular stress in the absence of a classical transcriptional UPR. However, ER-resident chaperones and oxidoreductases did interact with Tg^rdw^ and this might have been sufficient to prevent a UPR.

### The levels of chondrocyte apoptosis are normal, whereas proliferation is significantly decreased in the growth plates of *Col2-Tg*^rdw^ mice

**Fig. 5.**
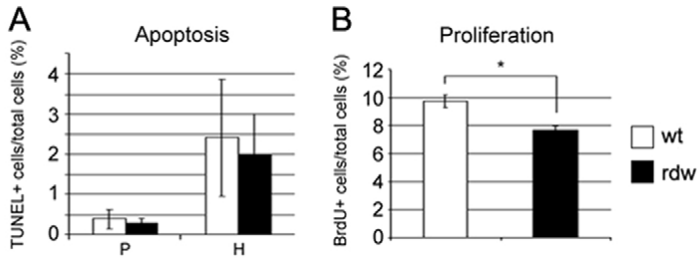
**Apoptosis is not induced, but chondrocyte proliferation is significantly reduced, in *Col2-Tg*^rdw^ transgenic mice.** (A) The rate of chondrocyte apoptosis in the proliferative (P) and hypertrophic (H) zones of the growth plate was analyzed by TUNEL and shown to be comparable between wild-type (wt; white columns) and *Col2-Tg*^rdw^ (rdw; black columns) mice. (B) Chondrocyte proliferation in the proliferative zone of the growth plate was significantly reduced by 21% (*P<0.01) in *Col2-Tg*^rdw^ mice (rdw; black columns) compared with wild-type (wt; white columns) mice.

The relative level of chondrocyte apoptosis was evaluated by TUNEL using proximal sections of the tibial growth plate of 3-week-old mice. TUNEL-positive cells were primarily localized at the vascular invasion front in both *Col2-Tg*^rdw^ and wild-type mice (our unpublished observations). The number of TUNEL-positive cells per total number of cells was comparable in the hypertrophic zone of *Col2-Tg*^rdw^ and wild-type mice (2.00±0.99% and 2.41±1.45%, respectively; *P*=0.71, three sections per mouse and three mice per genotype were analyzed). Furthermore, a similar limited number of TUNEL-positive cells were detected in the proliferative zone of both transgenic and wild-type animals (0.30±0.09% and 0.40±0.24%, respectively; *P*=0.81) (Fig. 5A).

The relative levels of chondrocyte proliferation were evaluated on the tibial growth plates of mice when growth rate is maximal at 3 weeks of age (Vanky et al., 1998). The proportion of BrdU-positive proliferative chondrocytes was significantly lower in *Col2-Tg*^rdw^ mice compared with controls (7.65±0.33% and 9.73±0.46%, respectively; *P*<0.01, three sections per mouse and three mice per genotype were analyzed), indicating that proliferation in the growth plates of transgenic mice was significantly reduced by 21% (Fig. 5B).

Microarray analysis of mRNA isolated from chondrocytes extracted from 5-day-old mice did not show any major differences in the relative expression of 21 genes associated with apoptosis. However, slight but statistically significant downregulation in the expression of mitogen-activated protein kinase kinase kinase 5 (*Map3k5*, −1.59-fold), nuclear factor of kappa light polypeptide gene enhancer in B-cells 1 (*Nfkb1*, −2.01-fold), tribbles homolog 3 (*Trib3*, −1.62-fold) and cach, cation transport-like regulator 1 (*Cach1*, −1.33-fold) was observed in *Col2-Tg*^rdw^ mice (supplementary material Table S3). In contrast, we confirmed the significant downregulation of several genes involved in cell proliferation, such as activin A receptor type 2 (*Acvr2a*, −4.00-fold), fibroblast growth factor 10 (*Fgf10*, −3.51-fold), fibroblast growth factor 1 (*Fgf1*, −4.13-fold), met proto-oncogene (*Met*, −5.44-fold) and nucleus accumbens associated 1 (*Nacc1*, −3.07-fold).

## DISCUSSION

Disturbed ECM networks, alterations in signalling pathways and intracellular stresses have each been suggested, either separately or in combination, as significant mechanisms contributing to disease pathogenesis in a number of different forms of chondrodysplasia (Cortes et al., 2009; Esapa et al., 2012; Forlino et al., 2005; Gualeni et al., 2010; Nundlall et al., 2010; Piróg-Garcia et al., 2007; Posey et al., 2009; Raducanu et al., 2009; Rajpar et al., 2009; Rodgers et al., 2007; Sahni et al., 2001; Wang et al., 2007; Wang et al., 2002). We have previously established the underlying importance of ER stress in the development of MCDS by targeting expression of the ER-stress-inducing *cog* mutant form of Tg exclusively to hypertrophic chondrocytes (Rajpar et al., 2009). In the current study we wished to determine the relative contribution of intracellular stress, induced predominantly in resting and proliferating chondrocytes, to growth plate pathology. A similar stress is sufficient to induce apoptosis and has been associated with severe pseudoachondroplasia (PSACH) caused by the *Comp* D469del mutation (Suleman et al., 2012). We therefore generated a transgenic mouse model expressing the apoptosis-inducing G2320R mutant form of Tg (Menon et al., 2007) under the collagen type II promoter (*Col2-Tg*^rdw^ mice).

*Col2-Tg*^rdw^ mice were either normal or slightly smaller at birth compared with wild type, but became visibly smaller than wild-type mice by 5 days of age. By 3 weeks of age transgenic animals were significantly lighter than wild-type controls and had developed an obvious short-limb dwarfism. As expected, intramembranous ossification was not affected in *Col2-Tg*^rdw^ mice. The delay in phenotypic presentation is consistent with several other targeted mouse models of chondrodysplasia in which mutant mice appear relatively normal at birth, but develop a short-limb dwarfism with increasing age (Cortes et al., 2009; Forlino et al., 2005; Piróg-Garcia et al., 2007; Posey et al., 2009; Rajpar et al., 2009; Sogawa et al., 2007; Suleman et al., 2012), and is also consistent with the clinical progression of the disease in some affected individuals. For example, individuals with PSACH and MED are normal at birth but start exhibiting progressive short-limb dwarfism during early childhood (Maroteaux and Lamy, 1959; Shivanand et al., 2007). Interestingly, from 3 weeks of age transgenic mice occasionally exhibited hip dysplasia, which is a feature reported in other mouse models of chondrodysplasia (Forlino et al., 2005; Piróg-Garcia et al., 2007; Rajpar et al., 2009; Rodgers et al., 2007; Suleman et al., 2012). However, this phenotypic feature was only present in a small proportion of *Col2-Tg*^rdw^ mice, suggesting variable expressivity.

The development of the growth plate was essentially normal in transgenic animals, with a typical vascular invasion front and characteristic formation of the secondary ossification centre. The resting, proliferating and hypertrophic zones of the growth plate were clearly distinguishable in *Col2-Tg*^rdw^ mice at 3 weeks of age, and the relative size of the different zones was normal. Interestingly, the growth plate in a small proportion of transgenic mice seemed to be more severely affected, showing disorganization to the columnar alignment of proliferating chondrocytes that could range from mild to severe. However, the level of growth plate disorganization did not correlate with the severity of the chondrodysplastic phenotype, confirming the variable expressivity of this pathological feature.

Proteomic analysis (co-immunoprecipitation and MS) confirmed that, in chondrocytes expressing Tg^rdw^, the mutant protein formed complexes with ER-resident chaperones and oxidoreductases; a similar observation was previously noted in thyrocytes (Menon et al., 2007). However, some of the interacting proteins were different from those observed in thyrocytes, suggesting cell-specific chaperone-foldase complexes. Conversely, no UPR or ERAD, or increased proteasomal degradation markers, were detected at the mRNA level at 5 days of age, suggesting that the apparent increase in the protein levels of ER chaperones and oxidoreductases might be due to increased protein half-life as previously reported (Menon et al., 2007) or their sequestration in mutant Tg complexes and/or aggregates, rather than to an increase in the relative expression of these genes. Indeed, under conditions of chronic ‘low-grade’ ER stress, BiP protein half-life is such that it does accumulate even under conditions in which transcription of BiP is not increased (Rutkowski et al., 2006). Alternatively, these specific chaperones and foldases might be translationally regulated, thereby influencing their relative levels in the mutant cell as a consequence of ER stress. For example, it has been suggested that cells can develop an adaptive response to mild ER stress through post-transcriptional and post-translational mechanisms (Rutkowski et al., 2006). Proteomic profiling of cartilage from 3-week-old mice identified at least 20 different chaperones and foldases; however, only the levels of calreticulin and peroxiredoxin 1 were significantly increased in mutant cartilage, suggesting that chondrocytes have adapted to the ER stress by this stage of the disease. A similar proteomic approach confirmed a robust UPR in chondrocytes expressing the V194D mutant form of matrilin-3, with significantly increased protein levels of BiP (Hspa5), Hsp47, PDIA1, PDIA3, PDIA4, PDIA6, Grp94 (Hsp90b1) and calreticulin (Bell et al., 2013).

Although our finding that the accumulation of mutant Tg^rdw^ did not induce a transcriptional UPR was somewhat unexpected, there have been other reports of novel stress pathways in models of human diseases that are UPR-independent. Indeed, we have recently shown that, in both cell and mouse models of PSACH caused by COMP D469del, there is no evidence of a UPR (Suleman et al., 2012), whereas cell models of osteogenesis imperfecta caused by triple helical mutations in type I collagen do not show increased levels of BiP (Chessler and Byers, 1992) and are proposed to induce an ‘aggregated protein response’ (APR) (Makareeva et al., 2011). Finally, models of serpinopathies in which the aggregation of misfolded insoluble α_1_-antitrypsin triggers an alternative ER stress response [termed ER overload response (EOR)] that is independent of the UPR but involves the activation of NF-κB signalling is another example (Ekeowa et al., 2010). Even though our approach relied upon the expression of an exogenous mutant protein, it nonetheless confirms that a transcriptional UPR is not the only response that might be induced by mutant protein retention and chronic ER stress. Understanding why different misfolded mutant proteins evoke different responses (i.e. UPR versus APR versus EOR) is an exciting new area of research in human pathobiology, and our recent finding that the consequences of individual mutations on protein misfolding and/or aggregation differs between *Matn3* V194D and *Comp* DelD469 mutant mice begins to provide a molecular explanation for why some misfolded mutant proteins induce a UPR and others do not (Bell et al., 2013).

Immunohistochemical analysis (IHC) confirmed that the retention of mutant Tg in the chondrocytes of *Col2-Tg*^rdw^ mice did not result in the co-retention of cartilage ECM molecules such as types II and X collagen, aggrecan and COMP, which were normally distributed within the ECM of transgenic mouse cartilage. For example, in *Col2-Tg*^rdw^ mice, type II collagen was localized predominantly to the ECM of the resting and proliferating zones, type X collagen was restricted to the hypertrophic zone, whereas aggrecan and COMP had the expected pericellular distribution. Moreover, the extractability of numerous ECM components was similar in *Col2-Tg*^rdw^ mice compared with wild-type cartilage, confirming that there was no major alteration to the ECM network at 3 weeks of age. The extractability of α1(II) collagen was slightly decreased in *Col2-Tg*^rdw^ cartilage following treatment with the low ionic buffer 1; however, this did not seem to have severe consequences on ECM composition as revealed by the normal distribution of type II collagen in *Col2-Tg*^rdw^ ECM following IHC. Furthermore, although α1(IX) collagen and osteomodulin showed increased extractability in buffer 1, the low number of spectral counts makes the biological relevance of these changes difficult to interpret. Finally, α3(VI) collagen and fibronectin showed increased extractability from mutant cartilage in buffer 2, suggesting that the pericellular matrix (PCM) of mutant chondrocytes might be slightly altered. Nevertheless, the extraction profile of other PCM-associated molecules is within normal limits with both the lower and higher ionic strength buffers, indicating that there are no major alterations to the pericellular environment.

Microarray experiments also showed no major alteration in the expression profile of cartilage-specific markers in *Col2-Tg*^rdw^ mice compared with wild-type animals at 5 days of age. This analysis provided further validation that the *Col2-Tg*^rdw^ transgenic mouse is a model of chondrodysplasia caused primarily by intracellular/ER stress without any major ECM alterations.

Increased and/or spatially dysregulated apoptosis and/or decreased chondrocyte proliferation have previously been ascribed as ‘core disease mechanisms’ in several mouse models of both phenotypically related and unrelated chondrodysplasias (Cortes et al., 2009; Forlino et al., 2005; Gualeni et al., 2010; Nundlall et al., 2010; Piróg-Garcia et al., 2007; Posey et al., 2009; Raducanu et al., 2009; Sahni et al., 2001; Suleman et al., 2012; Wang et al., 2007; Wang et al., 2002). We observed no increase or spatial dysregulation of chondrocyte apoptosis in the growth plates of *Col2-Tg*^rdw^ mice, which we have previously described in our mouse models of PSACH and MED (Leighton et al., 2007; Piróg-Garcia et al., 2007; Suleman et al., 2012). This finding was particularly surprising because the expression of Tg^rdw^ in the thyroid gland of *rdw/rdw* rats causes protein aggregation that leads to thyrocyte apoptosis (Menon et al., 2007). This observation therefore suggests that the expression levels of Tg^rdw^ in chondrocytes might not be high enough to induce apoptosis. Indeed, no increase in apoptosis was detected in the thyroid glands of *rdw/+* rats, suggesting that high levels of Tg^rdw^ expression are required to cause a cytotoxic effect (Menon et al., 2007). Interestingly, the expression levels of Tg^rdw^ in chondrocytes of *Col2-Tg*^rdw^ mice as evaluated by microarray analysis were comparable to those of matrilin-3. Previous studies have shown that the mechanism leading to MED caused by matrilin-3 mutations involves the induction of a UPR and dysregulated apoptosis (Leighton et al., 2007; Nundlall et al., 2010). Therefore the comparable level of Tg^rdw^ expression should be sufficient to modify apoptosis in growth plate chondrocytes. Alternatively, the lack of a pro-apoptotic effect of aggregated Tg^rdw^ in *Col2-Tg*^rdw^ mouse chondrocytes might be due to cell-specificity. Indeed, it is possible that some of the consequences of intracellular aggregation of Tg^rdw^ are cell-dependant and could differ between thyrocytes and chondrocytes.

Chondrocyte proliferation was reduced by 21% in *Col2-Tg*^rdw^ mice compared with wild-type controls. A similar reduction was found in various mouse models of PSACH and MED, which showed reduced proliferation rates of 17% (*Matn3* V194D) (Leighton et al., 2007), 18% (*Comp* D469del) (Suleman et al., 2012) and 26% (*Comp* T585M) (Piróg-Garcia et al., 2007). Therefore, although the expression of mutant Tg in *Col2-Tg*^rdw^ mouse chondrocytes was not sufficient to induce a significant UPR (compared with thyrocytes), it is nonetheless sufficient to produce a direct effect on chondrocyte proliferation.

Long bone growth occurs through a highly coordinated process involving proliferation, hypertrophy and finally apoptosis of terminal hypertrophic chondrocytes at the vascular invasion front. Although the largest contribution to long bone growth has been ascribed to the dramatic increase in volume of hypertrophic chondrocytes (Breur et al., 1991), this process is still dependent upon sufficient chondrocytes entering hypertrophy after proliferation. It therefore follows that reduced chondrocyte proliferation will have a profound effect on hypertrophy and indeed our study suggests that reduced proliferation is sufficient on its own to cause short-limb dwarfism. To further support this hypothesis, morphometric analysis did not demonstrate any differences in the overall width of the growth plate or in the spatial organization of the different zones, whereas the number of hypertrophic cells per chondrocyte column was comparable between genotypes.

Finally, as a possible consequence of reduced chondrocyte proliferation in *Col2-Tg*^rdw^ mice, we also observed areas of hypocellularity in the growth plate, a feature common to other mouse models of chondrodysplasia (Baradaran-Heravi et al., 2012; Leighton et al., 2007; Pfander et al., 2004; Piróg-Garcia et al., 2007; Suleman et al., 2012; Zaucke and Grässel, 2009), which has previously been attributed to increased and/or dysregulated apoptosis.

In summary, the data presented in this paper demonstrate that mutant protein retention and intracellular stress, without alterations to the secretion and assembly of the ECM, can directly cause chondrodysplasia in mice by reducing chondrocyte proliferation in the epiphyseal growth plate. We propose that reduced chondrocyte proliferation alone is sufficient to cause reduced long bone growth independently of increased and/or spatially dysregulated chondrocyte apoptosis. These important findings will significantly increase our understanding of the relative contributions of various disease mechanisms to the initiation and progression of chondrodysplasia. Furthermore, our study will help in delineating specific disease mechanisms that will pave the way for the identification of relevant therapeutic targets that influence detrimental changes in chondrocyte phenotype.

## MATERIALS AND METHODS

### Generation of *Col2-Tg*^rdw^ transgenic mice and genotyping by PCR and real-time qPCR

The mouse collagen type II (*Col2*) promoter cloned into pBluescript was a kind gift from Dr Attila Aszodi (LMU, Munich). The 8.5-kb cDNA encoding the *rdw* mutant form of Tg, including the start codon, three contiguous Myc tags and the polyadenylation site (Lee et al., 2009), was subcloned into an *Eco*RV site within the 3′ end of the *Col2* promoter. The construct was then removed from the vector by digestion with *Bss*HII and the DNA was purified for pronuclear injection into fertilized oocytes collected from FVB mice, which were subsequently implanted into pseudo-pregnant foster mothers.

The resulting offspring were assessed for the presence of the transgene by PCR between the *Col2* promoter and the *Tg*^rdw^ cDNA using the following primers: *Col2*-Gen forward: 5′-GCACCGTTCTCATGTGCAGG-3′ and *Tg*^rdw^-Gen reverse: 5′-TTCCATCTTCAGAGCACTGG-3′. Chimeras that were positive for the transgene (showing a band at ∼360 bp) were mated with wild-type C57BL/6 mice, and heterozygous F1 offspring were mated together to generate wild-type, heterozygous and homozygous mice. Heterozygous and homozygous genotypes were determined by quantitating the relative levels of the transgene to that of the type X collagen gene (*Col10a1*) using real-time qPCR on genomic DNA with the following primers: *Col2*-Rt forward: 5′-CATTCTTGGAGAACGCAGG-3′; *Tg*^rdw^-Rt reverse: 5′-ATGTTGGCTGCTACCAGG-3′; *Col10a1*-Rt forward: 5′-CTTCCTGTCAAGCTCATCC-3′; *Col10a1*-Rt reverse: 5′-TAGGATTGCTGAGTGCTCC-3′. Wild-type genomic DNA was used as a control and a ‘no template’ control was included to monitor contamination. All samples were run in duplicate using the SYBR^®^ Green (Applied Biosystems) PCR protocol on a Chromo4™ real-time PCR system (Bio-Rad). Only wild-type and homozygous animals were subsequently used in this study.

### Growth curves, X-rays and bone length measurements

Mice were weighed at 3, 6 and 9 weeks of age, and these values were used to generate growth curves. Mice were also radiographed at 3, 6 and 9 weeks of age using an X-ray specimen radiography system (Faxitron) and X-ray films (Amersham Hyperfilm). Individual bone lengths were measured from scanned radiographic images using propriety software (Certus Technology Associates Limited, Exeter, UK).

### Histology and IHC of fixed tissues

Hind limbs were dissected from wild-type and transgenic mice. Bones were cleaned of surrounding soft tissues and then fixed at room temperature overnight in either 4% paraformaldehyde (PFA, Sigma) or in a solution of 5% acetic acid in ethanol. Bone decalcification was performed in 20% EDTA at pH 7.4. Samples were then rinsed in distilled water, dehydrated through a series of increasing alcohols, cleared in xylene and embedded in paraffin wax. Sagittal sections 5 μm thick were cut using an HM 355S microtome (MicRom), collected on positively charged superfrost slides (VWR) and dried overnight prior to classical histological staining or IHC. For haematoxylin and eosin staining, slides were de-waxed in xylene, rehydrated through a series of decreasing alcohols, stained, dehydrated and cleared in xylene using an automatic stainer (ThermoShandon Ltd) prior to mounting using the xylene-based mounting medium Pertex (Leica). For 5-bromo-2′-deoxyuridine (BrdU) IHC, 3-week-old mice were injected intraperitoneally with 10 μl BrdU labeling solution/g body weight (Amersham) 2 hours before sacrifice. IHC using anti-Myc-tag antibody clone 4A6 (Millipore, #16–213, 1:300), anti-collagen II antibody (Abcam, #ab54236, prediluted), anti-collagen X antibody (Rajpar et al., 2009), anti-COMP antibody (Genetex, #GTX14515, 1:500) and anti-BrdU antibody (Invitrogen, #B35138, prediluted) was performed on 5% acetic acid in ethanol-fixed samples, whereas IHC using anti-aggrecan antibody (Santa Cruz Biotechnology, sc-25674, 1:25) and DeadEnd™ fluorometric terminal deoxynucleotidyl transferase dUTP nick end labelling (TUNEL) assay (Promega) for the detection of apoptotic cells were performed on PFA-fixed samples. After staining, microscopical images of the samples were collected using the Axiovision microscope software (Zeiss).

### Chondrocyte isolation, SDS-PAGE and western blotting of chaperones

Cartilage was dissected from the ribs of 5-day-old animals as previously described (Nundlall et al., 2010) and digested to release the chondrocytes. Chondrocytes were then passed through a 70-μm cell strainer, washed in Dulbecco’s modified Eagle’s medium (DMEM) containing 20% foetal bovine serum (FBS), centrifuged for 5 minutes at 350 ***g***, washed in phosphate buffered saline (PBS), centrifuged for 5 minutes at 350 ***g*** and re-suspended in 1 ml of PBS for cell counting. Aliquots of 10^5^ cells were re-suspended in 20 μl of 1× SDS-PAGE buffer, either with the addition of 5 μl of 1M DTT (reduced samples) or not (unreduced samples) and incubated at 95°C for 5 minutes prior to loading on 4–12% SDS-PAGE gels. The gel was electroblotted onto a nitrocellulose membrane, which was blocked with 5% skimmed milk powder in 1% Tween in PBS either at room temperature for 1 hour or at 4°C overnight. Protein detection was then performed using the antibodies anti-Myc-tag clone 4A6 (Millipore, #16–213, 1:2000), anti-ERp57 (Abcam, #27985, 1:500), anti-ERp72 (Santa Cruz, #G2506, 1:500), anti-PDI (Enzo, #ADI-SPA-890, 1:500) and anti-GAPDH (Millipore, MAB374, 1:500). When required, the blots were quantified by densitometry analysis using the ImageJ software (Abramoff et al., 2004) and normalized to GAPDH.

### Microarray hybridization

RNA was isolated from the rib chondrocytes of 5-day-old mice, as previously described (Nundlall et al., 2010). RNA extracted from the mice of three litters (on average eight pups per litter) of the same genotype was pooled and used for microarray analysis. RNA quality was tested using the RNA 6000 Nano Assay and analyzed using the Agilent 2100 Bioanalyser (Agilent Technologies). RNA concentration was assessed using the Nanodrop ultra low volume spectrophotometer (Nanodrop Technologies). The hybridization cocktail was hybridized to the Mouse 430_2 oligonucleotide array (Affymetrix). Arrays were read, processed and analyzed as previously described (Suleman et al., 2012).

### Real-time qPCR

mRNA was isolated from the rib chondrocytes of 5-day-old mice as previously described (Nundlall et al., 2010). mRNA extracted from three separate litters per genotype (on average eight pups per litter) was retrotranscribed to cDNA using random hexamer primers (Superscript III, Invitrogen), and real-time qPCR was performed using the SYBR^®^ Green (Applied Biosystems) PCR protocol on a Chromo4™ real-time PCR system (Bio-Rad). Primer sequences were: *Grp78/BiP* forward 5′-GGCACCTTCGATGTGTCTCTTC-3′; *Grp78/BiP* reverse 5′-TCCATGACCCGCTGATCAA-3′; *Grp94* forward 5′-TAAGCTGTATGTACGCCGCGT-3′; *Grp94* reverse 5′-GGAGATCATCGGAATCCACAAC-3′; *Cnx* forward 5′-TGATTTCCTCTCCCTCCCCTT-3′; *Cnx* reverse 5′-CACTGGAACCTGTTGATGGTGA-3′; *Crt* forward 5′-GCTACGTGAAGCTGTTTCCGA-3′; *Crt* reverse 5′-ACATGAACCTTCTTGGTGCCAG-3′; *Erp72* forward 5′-AGTATGAGCCCAGGTTCCACGT-3′ and *Erp72* reverse 5′-AGAAGTCTTACGATGGCCCACC-3′. Each experiment included ‘no template’ controls, was run in duplicate, and had an 18S RNA control (*18S* forward 5′-GTAACCCGTTGAACCCCATT-3′; *18S* reverse 5′-CCATCCAATCGGTAGTAGCG-3′).

### Immunoprecipitation of Myc-tagged Tg and interacting proteins

For immunoprecipitation experiments, cartilage was dissected from the ribs of 5-day-old animals as previously described (Nundlall et al., 2010) and digested to release the chondrocytes. Chondrocytes were then passed through a 70-μm cell strainer, washed in DMEM containing 20% FBS, centrifuged for 5 minutes at 350 ***g***, washed in PBS, centrifuged for 5 minutes at 350 ***g*** and incubated in 25 mM N-ethylmaleimide (NEM) in PBS for 20 minutes on ice in order to preserve protein-protein interaction. Cells were then centrifuged at 350 ***g*** for 5 minutes, washed in PBS and centrifuged again at 350 ***g*** for 5 minutes before being lysed for 5 minutes on ice in 25 mM Tris-HCl, pH 7.4, 150 mM NaCl, 1 mM EDTA, 1% Triton X-100, 5% glycerol, 0.5 mM PMSF. Cell lysates were centrifuged at 13,600 ***g*** for 10 minutes at 4°C and protein content was assessed on the supernatants using the BCA assay. The ProFound c-Myc Tag IP/Co-IP Kit (Thermo Scientific) was used according to the manufacturer’s instructions to precipitate the mutant Myc-tagged Tg together with interacting proteins.

To confirm that the protein of interest was correctly precipitated, a control western blotting experiment was performed. Samples were then loaded onto 10% SDS-PAGE gels and run into the gels for 3 minutes at 220 V. Gels were stained for 5 minutes with Instant*Blue* Coomassie-based stain (Expedeon) and washed with distilled water overnight. MS was used to identify the co-precipitated proteins interacting with mutant Tg.

### Sequential protein extractions of mouse cartilage

The femoral heads were dissected from 3-week-old mice, snap frozen in liquid nitrogen and stored at −80°C. On the day of the extraction, samples were thawed, roughly cut into small pieces and precisely weighted. Sequential extraction of the proteins was performed as described elsewhere (Nicolae et al., 2007). The first extraction was performed in 0.15 M NaCl, 50 mM Tris, pH 7.4 (buffer 1) with EDTA-free protease inhibitors (Roche); the second extraction was performed in 1 M NaCl, 10 mM EDTA, 50 mM Tris, pH 7.4 (buffer 2) with EDTA-free protease inhibitors; and the third extraction was performed in 4 M GuHCl, 10 mM EDTA, 50 mM Tris, pH 7.4 (buffer 3) with EDTA-free protease inhibitors. The extracted proteins were ethanol precipitated, air dried, re-suspended in SDS-PAGE sample buffer with 0.1 M DTT and boiled at 95°C for 5 minutes.

### Mass spectrometry (LC-MS/MS) analysis of cartilage extractions

Aliquots (20 μl) of femoral head cartilage extracts were run on 4–12% SDS-PAGE for 4 minutes (at 200 V). Total protein pools were cut from the gel before dehydration, reduction, alkylation and washing. Samples were digested overnight with trypsin at 37°C and analyzed by LC-MS/MS with a NanoAcquity LC (Waters, Manchester, UK) and LTQ Velos (Thermo Fisher Scientific, Waltham, MA) mass spectrometer. Peptides were concentrated on a pre-column (20 mm×180 μm i.d., Waters) and separated using a gradient from 99% A (0.1% formic acid in water) and 1% B (0.1% formic acid in acetonitrile) to 25% B, in 45 minutes at 200 nl/minute, using a 75 mm×250 μm i.d. 1.7 μm BEH C18, analytical column (Waters). Peptides were selected for fragmentation automatically by data-dependent analysis.

### Bioinformatic processing of LC-MS/MS data

Data were interrogated using Mascot version 2.2 (Matrix Science, UK) against the UniProt database (version 2011-05) with taxonomy of *Mus musculus* and the following search parameters selected: fragment tolerance: 0.6 Da; parent tolerance: 0.5 Da; fixed modifications allowed: +57 on C (carbamidomethyl), +16 on M (oxidation); max missed cleavages: 1. Mascot search results were validated using Scaffold version 3.3.1 (Proteome Software, Portland, OR) to assign confidence values to peptide/protein matches, where Peptide/Protein Prophet algorithm confidence values of 0.7 and 0.99 were used, respectively. Identified proteins were defined as having a number of matched peptide spectra ≥2, and the unweighted spectral count was used as a measure of quantification. These parameters constrained the protein false discovery rate (FDR) to ≤0.2% in all analyses. Three biological replicates were used in all experiments. The number of spectra identified for each protein was compared between wild-type and *Col2-Tg*^rdw^ mice using the beta-binomial test in R (version 2.14.2, BetaBinomial package). A *P*-value <0.05 was considered significant.

### General statistical analysis

Where applicable, statistical differences between the different groups tested were evaluated using Student’s *t*-test and results were expressed as means ± s.e.m. A value of *P*<0.05 was considered statistically significant.

## Supplementary Material

Supplementary Material
