# Supplementary material for: A novel transgenic mouse model of growth plate dysplasia reveals that decreased chondrocyte proliferation due to chronic ER stress is a key factor in reduced bone growth
Source: Dis Model Mech. 2013 Sep 12;6(6):1414–25. doi: 10.1242/dmm.013342 (PMC3820264; doi:10.1242/dmm.013342)
Supplement: Supplementary Material [file supp_6_6_1414__index.html]

A novel transgenic mouse model of growth plate dysplasia reveals that decreased chondrocyte proliferation due to chronic ER stress is a key factor in reduced bone growth — A novel transgenic mouse model of growth plate dysplasia reveals that decreased chondrocyte proliferation due to chronic ER stress is a key factor in reduced bone growth — Supplementary Material 

# A novel transgenic mouse model of growth plate dysplasia reveals that decreased chondrocyte proliferation due to chronic ER stress is a key factor in reduced bone growth

## DMM013342 Supplementary Material

**Files in this Data Supplement:**

- **Supplementary Material PDF**
