# Supplementary material for: A novel transgenic mouse model of growth plate dysplasia reveals that decreased chondrocyte proliferation due to chronic ER stress is a key factor in reduced bone growth
Source: Dis Model Mech. 2013 Sep 12;6(6):1414–25. doi: 10.1242/dmm.013342 (PMC3820264; doi:10.1242/dmm.013342)
Supplement: Supplementary Material [file supp_013342_DMM013342.pdf]

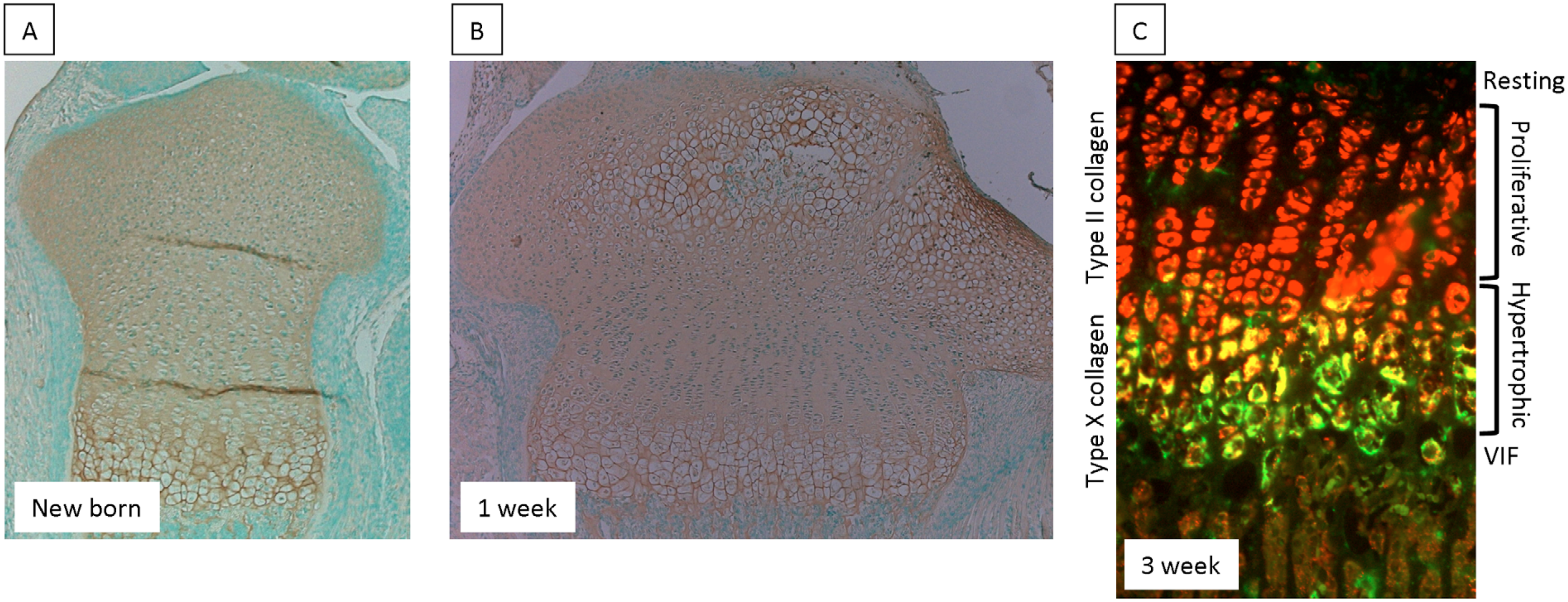

Supplemental Figure S1. Expression of type II and type X collagen in the cartilage growth plate. Immunohistochemistry (IHC) was used to demonstrate the expression of type II collagen at new born and at 1 week of age. *In situ* hybridization was used to demonstrate the expression of type II and type X collagens at 3 weeks of age. **A, B**) Type II collagen protein is present throughout the entire growth plate and articular cartilages (brown staining) at new born and 1 week of age. **C**) *In situ* hybridization for type II (red) and type X (green) collagen mRNA clearly shows that type II collagen is expressed predominantly in the resting and proliferative zones, whereas type X collagen expression is restricted to hypertrophic chondrocytes. Collagen II and X are both expressed in pre-hypertrophic chondrocytes (yellow), prior to the onset of hypertrophy and eventually apoptosis at the vascular invasion front (VIF).

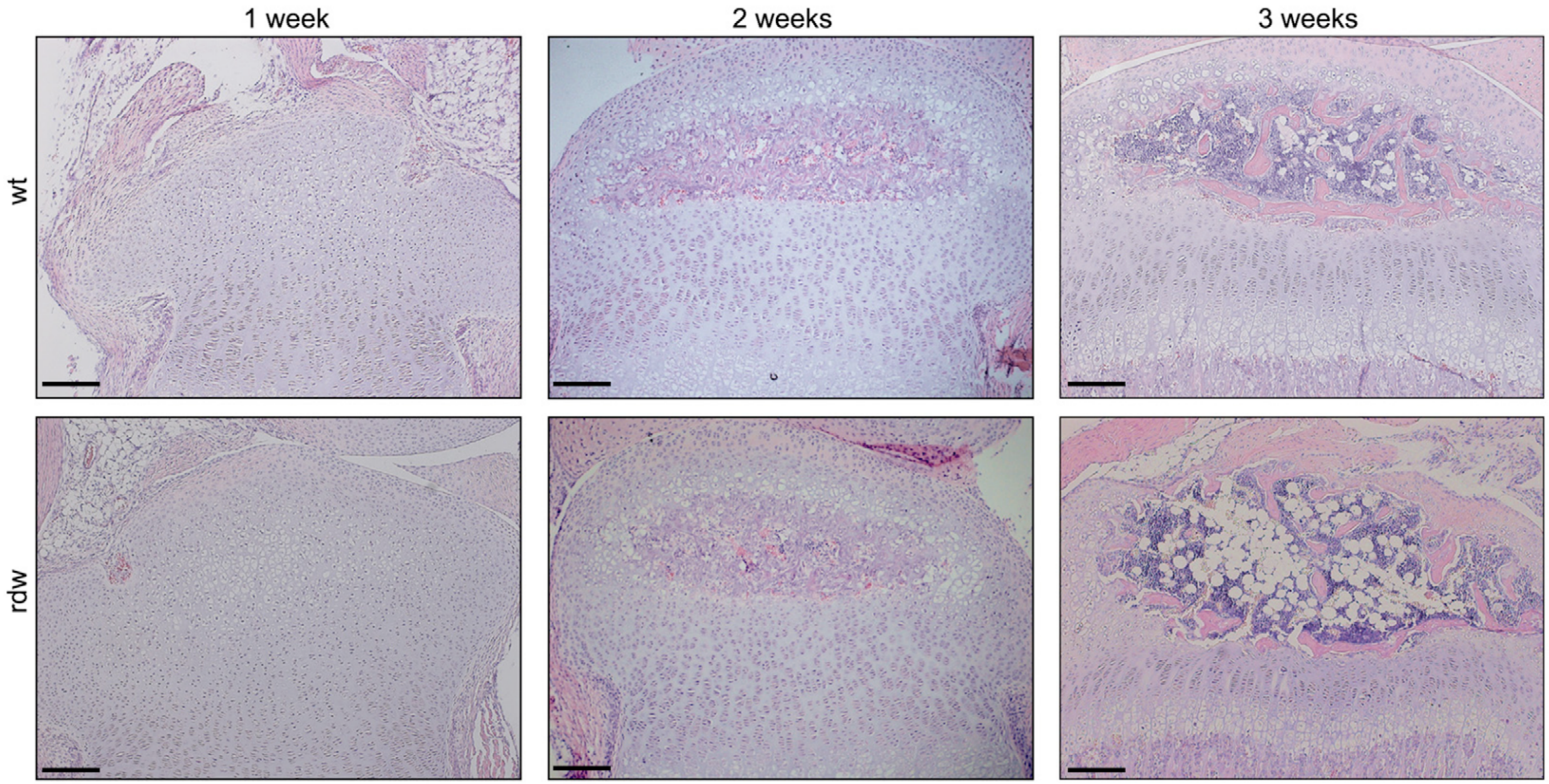

Supplemental Figure S2. Overall development of the growth plate and secondary centre of ossification is normal. H & E staining showing the development of the growth plate and formation of the secondary centre of ossification from 1 to 3 weeks of age. Wild-type (wt) and *Col2-Tg*^{rdw} (rdw) growth plates are comparable with clearly distinguishable resting, proliferating and hypertrophic zones. Black scale bars = 100 μm.

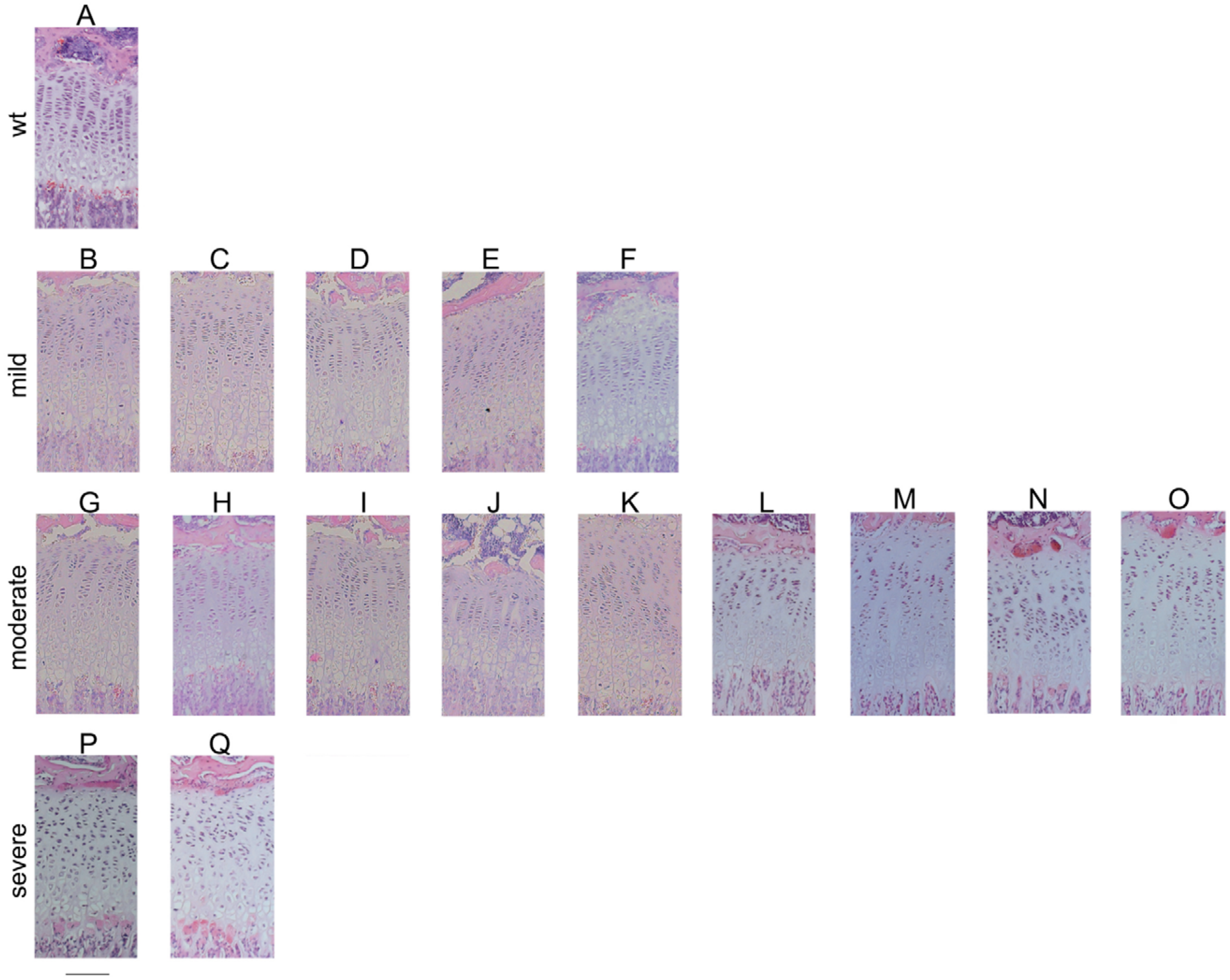

Supplemental Figure S3. Variable disorganisation of the growth plate in rdw mutant mice.
H & E staining demonstrating disorganisation of the proliferative zone in a proportion of *Col2-Tg* mutant mice compared to wild type (**A**). The disorganisation varied from mild (**B to F**), moderate (**G to O**) to severe (**P & Q**) and is characterised by the misalignment of chondrocytes within individual chondrons and a further disorganisation of chondrons within vertical stacks. In the milder cases, columnar alignment of chondrocytes is preserved, but larger areas of hypocellularity are observed. In severe cases, columnar alignment is partially lost. However, no correlation was observed between the grade of growth plate abnormality and the severity of the chondrodysplastic phenotype in mice. Black scale bar = 100 μm.

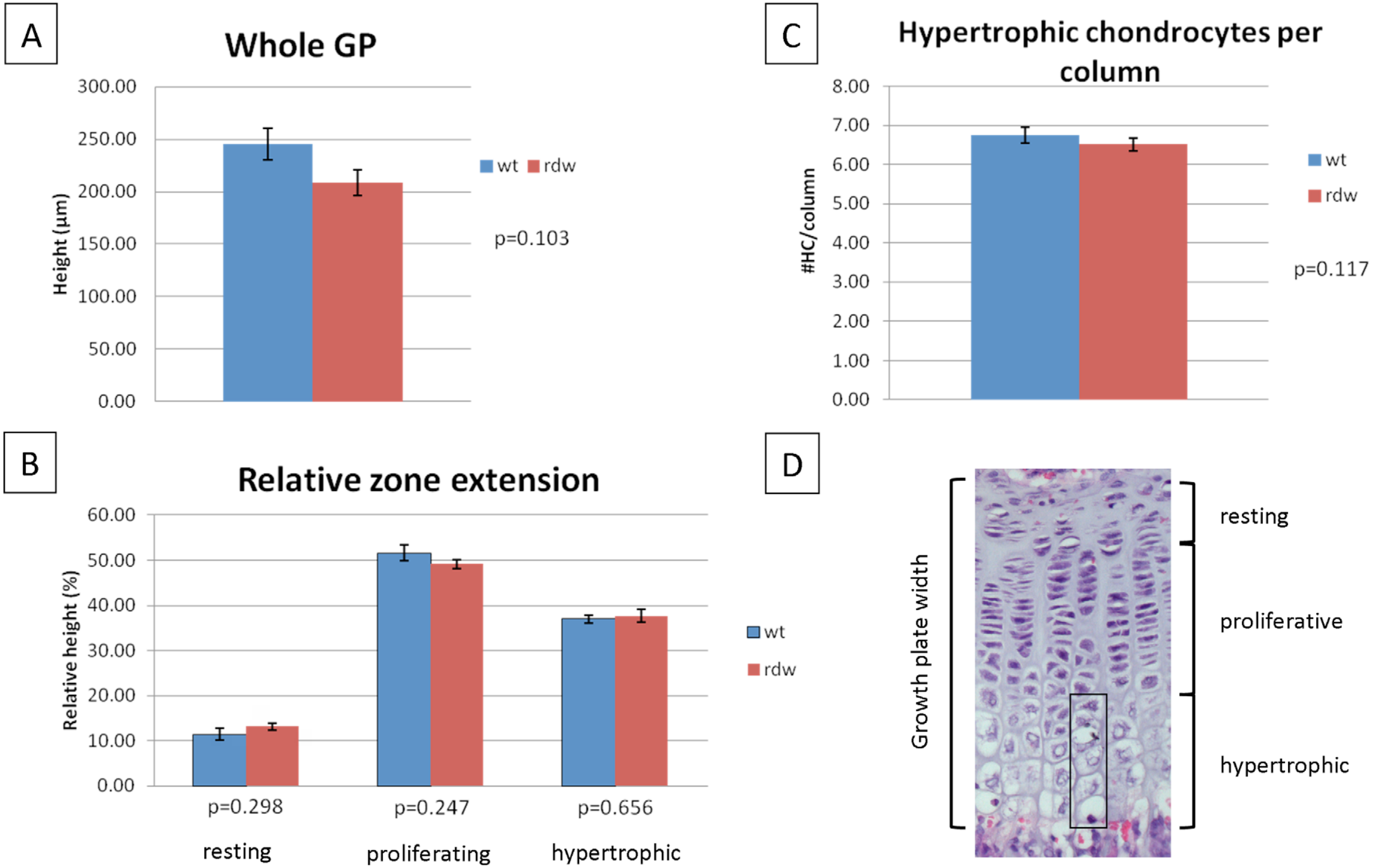

Supplemental Figure S4. Morphometric analysis of the cartilage growth plate from wild type and *Col2-Tg*^{rdw} mice at 3 weeks of age. A) Height (mm) of the entire growth plate shows no differences between wild type (wt) and *Col2-Tg*^{rdw} mice (rdw) mice. **B)** The relative proportions (%) of the individual resting, proliferative and hypertrophic zones were also comparable between wt and rdw mice. **C)** There were no differences in the number of hypertrophic chondrocytes in individual cell stacks within the hypertrophic zone. **D)** Schematic showing the various zones of the growth plate that were used for the morphometric analysis, whilst the boxed area shows a typical stack of hypertrophic cells.

Mouse	Gene	Mutation	Birth	Onset	Reduction in bone length			Cell Proliferation	Apoptosis	Micro array	Proteomics	Reference
					3 week	6 week	9-10 week	3 week	3 week			
EDM5	*Matn3*	V194D	Normal	2 week[1]	↓12% (tibia)	13% (tibia)	12% (tibia)	↓16%	Spatially dysregulated	5 day	3 week	Leighton 2007<br>Nundlall 2010<br>Bell 2013
PSACH	*Comp*	D469del	Normal	6 week[2]	No difference	↓6% (tibia)	↓6% (femur)<br>↓5% (tibia)	↓17%	↑90-fold (Pz)<br>↑5-fold (Hz)	NB<br>5 day<br>2 & 3 week	3 week	Suleman 2012<br>Bell 2013
EDM1	*Comp*	T585M	Normal	9 week[3]	No difference	No difference	↓6% (tibia)	↓24%	↑3-fold (Rz)<br>↑12-fold (Pz)<br>↑3-fold (Hz)	5 day	3 week	Pirog-Garia 2007<br>Bell 2013
RDW	*Col2-TG_RDW*	G2320R	Slightly smaller	Birth[4]	↓9% (femur)<br>↓8% (tibia)	↓6% (femur)<br>↓5% (tibia)	↓8% (femur)<br>↓5% (tibia)	↓21%	No difference	5 day	3 week	This study
MCDS	*Col10a1*	N617K	Normal	1 week[5]	↓16% (femur)	Not performed	↓20% (femur)	No difference	No difference	2 week	Not performed	Rajpar 2010<br>Cameron 2011
Cog	*ColX-TG_Cog*	L2263P	Normal	1 week[6]	↓4% (femur)	↓8% (femur)	Not performed	No difference	No difference	2 week	Not performed	Rajpar 2010<br>Cameron 2011

Table S1. Phenotypic consequences in targeted mouse models and novel phenocopies of epiphyseal and metaphyseal dysplasias.
An overview of the phenotype and pathological features in mouse models of epiphyseal (EDM5, PSACH, EDM1, RDW) and metaphyseal (MCDS, Cog) dysplasia and for each mouse model relevant genes and mutations are indicated with the phenotype at birth and/or age of onset of symptoms (i.e. short limbs). A significant reduction in cell proliferation and changes in apoptosis are shown at 3 weeks of age. Microarray and proteomics studies were primarily performed at 5 days and 3 weeks of age respectively. Key: NB = new born; Rz = resting zone; Pz = proliferative zone; Hz = hypertrophic zone.

1. Mutant mice are normal at birth but significant reduction in length of tibia occurs by 2 weeks of age.
2. Mutant mice are normal at birth but significant reduction in length of tibia occurs by 6 weeks of age.
3. Mutant mice are normal at birth but significant reduction in length of tibia occurs by 9 weeks of age.
4. Mutant mice were slightly shorter that wild type littermates at birth and a significant reduction in the length of tibia and femur occur by 9 weeks of age.
5. Mutant mice are normal at birth but significant reduction in length of femur occurs by 2 weeks of age.
6. Mutant mice are normal at birth but significant reduction in length of femur occurs by 1 weeks of age and newborn mutant mice exhibited an expanded hypertrophic zone.

**Mouse Genotype**	**WT**			**RDW**				**WT**			**RDW**				**WT**			**RDW**			**V194D**	**T585M**	**D469Del**
**Buffer**	**Buffer 1**			**Buffer 1**				**Buffer 2**			**Buffer 2**				**Buffer 3**			**Buffer 3**					
**Replicate number**	**1**	**2**	**3**	**1**	**2**	**3**	**n.s**	**1**	**2**	**3**	**1**	**2**	**3**	**n.s**	**1**	**2**	**3**	**1**	**2**	**3**			
α1(XII) collagen	24	18	13	21	23	22	n.s	27	29	30	17	41	28	n.s	18	20	25	23	26	26	↓*[2]	↓*[2]	n.s
α 1(II) collagen	14	11	18	11	8	9	↓*	22	19	19	16	14	21	n.s	16	13	13	12	17	12	n.s	n.s	n.s
α 3(VI) collagen	4	3	3	4	4	0	n.s	2	2	0	3	8	6	↑*	35	26	38	30	35	39	↑*[3]	n.s	n.s
α 2(XI) collagen	6	3	6	6	4	7	n.s	13	14	14	9	9	10	n.s	6	8	4	5	7	10	n.s	n.s	n.s
α 2(XI) collagen	6	3	6	6	4	7	n.s	13	14	14	9	9	10	n.s	6	8	4	5	7	10	n.s	n.s	n.s
α1(I) collagen	2	5	5	5	6	4	n.s	2	3	0	0	2	0	n.s	6	6	3	4	6	6	n.s	n.s	↑**[2]
α1(XIV) collagen	5	10	7	4	11	0	n.s	0	6	7	4	14	0	n.s	0	0	0	0	4	0	↓*[1,2]	↑*[3]	↓**[1,2]
α1(XI) collagen	7	5	7	6	4	5	n.s	7	8	8	8	6	7	n.s	5	5	7	3	4	7	n.s	↓*[1]	n.s
α1(IX) collagen	0	0	0	2	3	0	↑*	3	7	5	6	5	7	n.s	3	4	4	6	3	3	n.s	↓*[1]↑*[3]	↓**[1]
α2(I) collagen	6	0	2	5	2	3	n.s	0	0	4	0	0	0	n.s	4	5	4	6	7	4	n.s	n.s	n.s
Matrilin-1	8	4	6	6	3	6	n.s	27	26	25	20	21	23	n.s	26	18	20	20	20	21	n.s	n.s	n.s
Matrilin-3	4	5	4	3	4	4	n.s	13	11	7	9	9	6	n.s	10	7	6	9	13	11	↓***[2,3]	n.s	n.s
Matrilin-4	0	0	0	0	0	0	n.s	3	4	9	8	5	3	n.s	3	3	3	3	6	0	n.s	n.s	n.s
Aggrecan	14	7	10	17	9	8	n.s	8	8	12	9	11	12	n.s	25	19	25	18	18	19	n.s	n.s	n.s
Perlecan	0	0	0	0	0	0	n.s	5	0	3	3	10	5	n.s	33	35	30	41	22	30	n.s	n.s	n.s
Vimentin	13	14	23	17	13	19	n.s	4	2	8	3	4	5	n.s	25	22	23	21	19	21	n.s	↑**[2]	↑***[2]
Thrombospondin 1	9	7	7	8	8	5	n.s	19	20	22	19	16	18	n.s	15	19	11	15	19	9	n.s	n.s	n.s
COMP	12	9	14	19	9	10	n.s	16	16	14	13	12	17	n.s	11	9	13	12	12	11	n.s	n.s	n.s
Fibronectin	6	0	5	7	4	5	n.s	6	5	4	10	12	9	↑**	20	21	24	24	30	22	n.s	↓*[2]	n.s
Link protein	0	3	4	4	0	0	n.s	3	5	6	3	0	5	n.s	18	16	12	20	11	12	n.s	n.s	n.s
Fibromodulin	3	3	3	3	7	3	n.s	9	8	6	5	7	11	n.s	11	8	5	6	8	12	n.s	n.s	n.s
α-2-HS-glycoprotein	5	0	4	4	3	5	n.s	11	12	14	13	9	13	n.s	11	7	5	8	8	10	↓*[3]	↓*[3]	n.s
Osteomodulin	0	0	0	0	2	2	↑*	5	3	0	2	4	6	n.s	5	3	3	7	6	5	↑*[1]	n.s	n.s
Chondroadherin	4	5	4	7	4	4	n.s	5	8	7	3	7	7	n.s	12	9	6	9	6	9		n.s	n.s
Biglycan	2	0	2	2	2	0	n.s	3	3	4	2	6	8	n.s	15	18	13	11	11	12	↑*[1]	n.s	n.s

Osteonectin	4	3	6	6	6	3	n.s	11	10	11	12	9	9	n.s	4	3	3	2	3	7	n.s	n.s	n.s
Epiphycan	3	0	5	5	0	3	n.s	0	0	2	3	3	0	n.s	7	4	6	6	7	6	↓**[2]	n.s	↓**[2]
Decorin	3	3	3	6	2	4	n.s	3	5	0	2	2	5	n.s	8	10	6	3	9	11	↓*[2]	n.s	n.s
Tenascin X	0	0	0	0	0	0	n.s	6	2	4	0	24	3	n.s	8	0	5	7	10	10	n.s	n.s	↓**[3]
Tenascin C	0	0	0	0	0	0	n.s	0	0	0	0	0	0	n.s	5	4	9	9	8	7	↓**[3]	n.s	↓**[3]
Lumican	4	4	6	6	7	4	n.s	4	6	3	5	3	4	n.s	3	4	2	4	7	3	n.s	n.s	n.s
Nidogen-2	0	0	0	2	0	0	n.s	2	0	0	2	2	3	n.s	17	11	10	5	10	10	n.s	n.s	↓*[3]

Table S2. Extractability of extracellular matrix (ECM) proteins from the cartilage of wild-type and transgenic *Col2-Tg*^{rdw} mice.

Cartilage protein pools following extraction with buffers 1, 2 and 3 were analyzed by LC-MS/MS and the data evaluated using Mascot against the UniProt murine database and validated with Scaffold using peptide/protein confidence values of 0.95 and 0.99 respectively. Positively identified proteins were defined as those having a number of matched peptide spectra >2. Three biological replicates were used in all experiments and the number of matched spectra from each buffer/replicate is presented. For the composition of Buffer1, Buffer 2, and Buffer 3 please refer to the Materials and Methods section. Statistical significance was determined using the beta-binomial test: *p<0.05; **p<0.02; ***p<0.005. Key: WT = wild-type mice; RDW = *Col2-Tg*^{rdw} mice; Δ = relative difference of RDW/WT; ↑ = increased extraction; ↓ = decreased extraction; n.s. = not significant. Significant differences in the cartilage protein extraction profiles of 3 targeted mouse models are also presented as a comparison (from Bell et al., 2013). V194D = *Matn3* V194D; T585M = *Comp* T585M; D469del= *Comp* D469del and the relevant extraction buffers are indicated in by square brackets.

Table S3. The fold change in the relative expression levels of UPR, ERAD and cartilage-associated genes in *Col2-Tg*^{rdw} mice compared to wild-type mice at 5 days of age. RNA was isolated the rib chondrocytes of 5 day old wild-type and *Col2-Tg*^{rdw} mice. The relative expression levels of genes calculated using the Robust Multiarray Averaging (RMA) method are shown with the significance value given in parentheses. Highlighted in yellow are values that reach statistically significance difference compared to wild-type (i.e. P>0.95 for up regulated genes and P<0.05 for down regulated genes. The gene order and categorization of the genes is based on that published by Cameron et al., 2012.

Canonical ER Stress Sensors

NCBI	Gene Name	Gene Symbol	Synonyms	Relative Expression RDW vs WT (PPLR Value)
NM_001081304	Activating transcription factor 6	Atf6	Atf6a	-1.24 (0.311)
NM_023913	Endoplasmic reticulum to nucleus signalling 1	Ern1	Ire1, Ire1a, Ire1p	1.21 (0.436)
NM_010121	Eukaryotic translation initiation factor 2 alpha kinase 3	Eif2ak3	Perk, Pek	2.44 (0.593)

Targets of Canonical ER Stress Sensors

NCBI	Gene Name	Gene Symbol	Synonyms	Relative Expression RDW vs WT (PPLR Value)
Atf6:				
NM_009722	ATPase, Ca++ transporting, cardiac muscle, slow twitch 2	Atp2a2	Serca2	-1.21 (0.462)
NM_007837	DNA-damage inducible transcript 3	Ddit3	Chop, Chop10, Gadd153	-1.12 (0.284)
NM_022310	Heat shock protein 5	Hspa5	Bip, Grp78, Hsce70, Sez7	-1.12 (0.324)
NM_027400	Lectin, mannose-binding, 1	Lman1	Ergic53, F5f8d, gp58, Mcfd1, Mr60, P58	1.18 (0.774)
NM_029103	Mesencephalic astrocyte-derived neurotrophic factor	Manf	Armet	1.02 (0.562)
NM_008725	Natriuretic protein precursor type A	Nppa	Anp, Pnf, Pnd	1.19 (0.626)
NM_011850	Nuclear receptor subfamily 0, group b, member 2	Nr0b2	Shp, Shp1	-5.67 (0.172)
NM_008814	Pancreatic and duodenal homeobox 1	Pdx1	Idx1, Ipf1, Mody4, Stf1	-3.24 (0.267)
NM_028769	Synovial apoptosis inhibitor 1 (Synoviolin)	Syvn1	Hrd1	1.06 (0.611)
NM_020275	Tumor necrosis factor receptor superfamily, member 10b	Tnfrsf10b	Dr5, Cd262, Killer, Trick2, Trickb, Ztnfr9, Trailr2, Trick2a, Trick2b	-1.24 (0.157)
NM_013842	X-box binding protein 1	Xbp1	Treb5	-1.05 (0.331)
Eif2ak3:				
NM_007498	Activating transcription factor 3	Atf3	Lrg21	-1.26 (0.040)
NM_007837	DNA-damage inducible transcript 3	Ddit3	Chop, Chop10, Gadd153	-1.12 (0.284)
NM_001005509	Eukaryotic translation initiation factor 2a	Eif2a		-1.15 (0.227)
NM_026114	Eukaryotic translation initiation factor 2, subunit 1 alpha	Eif2s1	35kDa	-1.19 (0.186)
NM_023913	Endoplasmic reticulum to nucleus signalling 1	Ern1	Ire1, Ire1a, Ire1p	1.21 (0.436)
NM_022310	Heat shock protein 5	Hspa5	Bip, Grp78, Hsce70, Sez7	-1.12 (0.324)
NM_010481	Heat shock protein 9	Hspa9	74kDa, Csa, Grp75, Hsc74, Hsp74, Hsp74a, Hspa9a, Mortalin, Mot2, Nthso70, Pbp74	-1.46 (0.129)

NM_010512	Insulin-like growth factor 1	Igf1		1.92 (0.976)
NM_028782	Ion peptidase 1, mitochondrial	Lonp1	Prss15	1.18 (0.695)
NM_010902	Nuclear factor, erythroid derived 2, like 2	Nfe2l2	Nrf2	-1.36 (0.172)
NM_008689	Nuclear factor of light polypeptide gene enhancer in B-cells 1	Nfkb1	NF-kappaB, NF-kappaB1, NF-KB1, p105, p50, p50/p105	-2.10 (0.000)
NM_019408	Nuclear factor of light polypeptide gene enhancer in B-cells 2	Nfkb2	lyt, NF-kappaB2, p49, p49/p100, p50B, p52	-1.55 (0.085)
NM_008654	Protein phosphatase 1, regulatory (inhibitor) subunit 15a	Ppp1r15a	Gadd34, Myd116	-1.06 (0.300)
NM_001039521	Rrn3 RNA polymerase I transcription factor homolog (yeast)	Rrn3	Tif1a	-1.26 (0.059)
NM_011716	Wolfram syndrome 1 homolog	Wfs1	Wolframin	1.00 (0.496)

Ern1:

NM_001081304	Activating transcription factor 6	Atf6	Atf6a	-1.24 (0.311)
NM_007837	DNA-damage inducible transcript 3	Ddit3	Chop, Chop10, Gadd153	-1.12 (0.284)
NM_022310	Heat shock protein 5	Hspa5	Bip, Grp78, Hsce70, Sez7	-1.12 (0.324)
NM_011952	Mitogen-activated protein kinase 3	Mapk3	Erk1, Esrk1, p44, p44erk1, p44mapk, Prkm3	1.12 (0.714)
NM_016700	Mitogen-activated protein kinase 8	Mapk8	Jnk, Jnk1, Prkm8, Sapk1	-1.14 (0.259)
NM_008689	Nuclear factor of light polypeptide gene enhancer in B-cells 1	Nfkb1	NF-kappaB, NF-kappaB1, NF-KB1, p105, p50, p50/p105	-2.10 (0.000)
NM_019408	Nuclear factor of light polypeptide gene enhancer in B-cells 2	Nfkb2	lyt, NF-kappaB2, p49, p49/p100, p50B, p52	-1.55 (0.085)
NM_015760	NADPH oxidase 4	Nox4		1.00 (0.500)
NM_011951	Mitogen-activated protein kinase 14	Mapk14	p38, Crk1, Mxi2, p38a, Csbp2, Csbp1, Prkm14, Prkm15, p38Mapk, p38alpha	1.34 (0.563)
NM_028769	Synovial apoptosis inhibitor 1 (Synoviolin)	Syvn1	Hrd1	1.06 (0.611)
NM_013693	Tumor necrosis factor	Tnf	Dif, Tnfa, Tnfsf2, Tnfsf1a, Tnfalpha	-1.76 (0.127)
NM_020275	Tumor necrosis factor receptor superfamily, member 10b	Tnfrsf10b	Dr5, Cd262, Killer, Trick2, Trickb, Ztnfr9, Trailr2, Trick2a, Trick2b	-1.24 (0.157)
NM_011716	Wolfram syndrome 1 homolog	Wfs1	Wolframin	1.00 (0.496)
NM_013842	X-box binding protein 1	Xbp1	Treb5	-1.05 (0.331)

Additional ER Stress Sensors

NCBI	Gene Name	Gene Symbol	Synonyms	Relative Expression RDW vs WT (PPLR Value)
NM_030080	Androgen induced bZIP transcription factor	Aibzip	Creb3l4, Tisp40	-1.24 (0.287)
NM_178661	BBF2 human homolog on chromosome 7	Bbf2h7	Creb3l2	-1.09 (0.228)
NM_145365	cAMP responsive element binding protein 3-like 3	Crebh	Creb3l3	-1.06 (0.471)
NM_013497	cAMP responsive element binding 3	Luman	Creb3	1.30 (0.808)
NM_011957	Old astrocyte specifically induced substance	Oasis	Creb3l1	1.12 (0.605)

ERAD

NCBI	Gene Name	Gene Symbol	Synonyms	Relative Expression RDW vs WT (PPLR Value)

NM_007597	Calnexin	Canx	Cnx	1.00 (0.514)
NM_007591	Calreticulin	Calr	Crt	-1.05 (0.373)
NM_024207	Der1-like domain family, member 1 (Derlin 1)	Derl1	Derlin-1	1.11 (0.694)
NM_033562	Der1-like domain family, member 2 (Derlin 2)	Derl2	Derlin-2	1.37 (0.907)
NM_024440	Der1-like domain family, member 3 (Derlin 3)	Derl3	Derlin-3	1.49 (0.699)
NM_010072	Dolichol-phosphate (beta-D) mannosyltransferase 1	Dpm1		-1.68 (0.190)
NM_010073	Dolichol-phosphate (beta-D) mannosyltransferase 2	Dpm2		1.31 (0.989)
NM_138677	ER degradation enhancer, mannosidase alpha-like 1	Edem1		-1.17 (0.543)
NM_145537	ER degradation enhancer, mannosidase alpha-like 2	Edem2		1.09 (0.613)
NM_001039644	ER degradation enhancer, mannosidase alpha-like 3	Edem3		-1.06 (0.385)
NM_022331	Homocysteine-inducible, endoplasmic reticulum stress-inducible, ubiquitin-like domain member 1	Herpud1	Sup, Herp, Mifl	-1.15 (0.168)
NM_020586	HERPUD family member 2	Herpud2		1.25 (0.438)
NM_007952	Protein disulphide isomerase associated 3	Pdia3	Erp57, Erp60, Erp61, Grp58	1.00 (0.445)
NM_009787	Protein disulphide isomerase associated 4	Pdia4	Erp72, Cai	1.10 (0.723)
NM_028295	Protein disulphide isomerase associated 5	Pdia5	Pdir	1.16 (0.748)
NM_027959	Protein disulphide isomerase associated 6	Pdia6	CaBP5, p5	-1.19 (0.336)
NM_016906	Sec61 alpha subunit 1	Sec61a1		1.06 (0.596)
NM_021305	Sec61 alpha subunit 2	Sec61a2		-1.10 (0.286)
NM_008408	STT3, subunit of the oligosaccharyltransferase complex, homolog A	Stt3a	Itm1	1.02 (0.541)
NM_024222	STT3, subunit of the oligosaccharyltransferase complex, homolog A	Stt3b	Simp	1.69 (0.803)
NM_028769	Synovial apoptosis inhibitor 1 (Synoviolin)	Syvn1	Hrd1	1.06 (0.611)

Proteasomal subunits:

NCBI	Gene Name	Gene Symbol	Synonyms	Relative Expression RDW vs WT (PPLR Value)
NM_011965	Proteasome (prosome, macropain) subunit, alpha type 1	Psma1	C2, alpha-type	-1.26 (0.508)
NM_008944	Proteasome (prosome, macropain) subunit, alpha type 2	Psma2	Lmpc3	1.12 (0.821)
NM_011184	Proteasome (prosome, macropain) subunit, alpha type 3	Psma3	Lmpc8	1.04 (0.579)
NM_011966	Proteasome (prosome, macropain) subunit, alpha type 4	Psma4	C9	1.18 (0.993)
NM_011967	Proteasome (prosome, macropain) subunit, alpha type 5	Psma5	Zeta	1.16 (0.804)
NM_011968	Proteasome (prosome, macropain) subunit, alpha type 6	Psma6	Iota	1.07 (0.621)
NM_011969	Proteasome (prosome, macropain) subunit, alpha type 7	Psma7	C6-I	1.42 (0.964)
AK010717	Proteasome (prosome, macropain) subunit, alpha type 8	Psma8		-1.32 (0.256)

NM_011185	Proteasome (prosome, macropain) subunit, beta type 1	Psmb1	Lmpc5	-1.02 (0.500)
NM_011970	Proteasome (prosome, macropain) subunit, beta type 2	Psmb2	C7-I	1.17 (0.861)
NM_011971	Proteasome (prosome, macropain) subunit, beta type 3	Psmb3	C10-II	1.12 (0.788)
NM_008945	Proteasome (prosome, macropain) subunit, beta type 4	Psmb4		1.07 (0.672)
NM_011186	Proteasome (prosome, macropain) subunit, beta type 5	Psmb5		1.25 (0.915)
NM_008946	Proteasome (prosome, macropain) subunit, beta type 6	Psmb6	Mpnd, Lmp19	1.11 (0.800)
NM_011187	Proteasome (prosome, macropain) subunit, beta type 7	Psmb7	MC14	1.25 (0.829)
NM_010724	Proteasome (prosome, macropain) subunit, beta type 8	Psmb8	Lmp7	-1.11 (0.484)
NM_013585	Proteasome (prosome, macropain) subunit, beta type 9	Psmb9	Lmp2	-1.39 (0.433)
NM_013640	Proteasome (prosome, macropain) subunit, beta type 10	Psmb10	Mecl1	1.53 (0.670)
NM_008947	Protease (prosome, macropain) 26S subunit, ATPase 1	Psmc1	S4, P26s4	1.16 (0.837)
NM_011188	Protease (prosome, macropain) 26S subunit, ATPase 2	Psmc2		1.01 (0.519)
NM_008948	Protease (prosome, macropain) 26S subunit, ATPase 3	Psmc3	Tbp1	1.09 (0.689)
NM_011874	Protease (prosome, macropain) 26S subunit, ATPase 4	Psmc4	Cip21, Mip224	1.10 (0.684)
NM_008950	Protease (prosome, macropain) 26S subunit, ATPase 5	Psmc5	mSUG1	1.12 (0.696)
NM_025959	Protease (prosome, macropain) 26S subunit, ATPase 6	Psmc6		-1.19 (0.140)
NM_027357	Proteasome (prosome, macropain) 26S subunit, non-ATPase, 1	Psmd1	S1, P112	-1.13 (0.365)
NM_134101	Proteasome (prosome, macropain) 26S subunit, non-ATPase, 2	Psmd2	Tex190, Teg190	1.20 (0.594)
NM_009439	Proteasome (prosome, macropain) 26S subunit, non-ATPase, 3	Psmd3	Psd3, AntP91a, Tstap91a	1.02 (0.522)
NM_008951	Proteasome (prosome, macropain) 26S subunit, non-ATPase, 4	Psmd4	Af1, Mcb1	1.04 (0.546)
NM_080554	Proteasome (prosome, macropain) 26S subunit, non-ATPase, 5	Psmd5	S5b	1.29 (0.724)
NM_025550	Proteasome (prosome, macropain) 26S subunit, non-ATPase, 6	Psmd6		1.11 (0.685)
NM_010817	Proteasome (prosome, macropain) 26S subunit, non-ATPase, 7	Psmd7	Mov34	1.32 (0.687)
NM_026545	Proteasome (prosome, macropain) 26S subunit, non-ATPase, 8	Psmd8		-1.02 (0.436)
NM_026000	Proteasome (prosome, macropain) 26S subunit,	Psmd9	P27, Bridge-I	1.58 (0.708)

	non-ATPase, 9			
NM_016883	Proteasome (prosome, macropain) 26S subunit, non-ATPase, 10	Psmd10		1.31 (0.932)
NM_178616	Proteasome (prosome, macropain) 26S subunit, non-ATPase, 11	Psmd11	S9	-1.28 (0.326)
NM_025894	Proteasome (prosome, macropain) 26S subunit, non-ATPase, 12	Psmd12	P55	-1.07 (0.264)
NM_011875	Proteasome (prosome, macropain) 26S subunit, non-ATPase, 13	Psmd13	S11	1.34 (0.798)
NM_021526	Proteasome (prosome, macropain) 26S subunit, non-ATPase, 14	Psmd14	Pad1, Poh1, rpm11	1.04 (0.662)
NM_011189	Proteasome (prosome, macropain) 28S subunit, alpha	Psme1	PA28a	1.01 (0.513)
NM_011192	Proteasome (prosome, macropain) 28S subunit, 3	Psme3	Ki, REGgamma, pa28g	1.07 (0.414)
NM_134013	Proteasome (prosome, macropain) 28S subunit, 4	Psme4	TEMO	-1.24 (0.231)
NM_212446	Proteasome (prosome, macropain) inhibitor subunit 1	Psmf1	PI31	1.11 (0.690)

Molecular Chaperones:

NCBI	Gene Name	Gene Symbol	Synonyms	Relative Expression RDW vs WT (PPLR Value)
NM_008298	DnaJ (Hsp40) homolog, subfamily A, member 1	Dnaja1	Hsj2, Nedd7	1.06 (0.650)
NM_019794	DnaJ (Hsp40) homolog, subfamily A, member 2	Dnaja2	Dnaj, Dnj3, mDj3, Dnaj3, Hirip4	-1.25 (0.124)
NM_023646	DnaJ (Hsp40) homolog, subfamily A, member 3	Dnaja3	Tid1, Tid1l	1.78 (0.103)
NM_021422	DnaJ (Hsp40) homolog, subfamily A, member 4	Dnaja4	Dj4, Hsj4	-1.15 (0.316)
NM_018808	DnaJ (Hsp40) homolog, subfamily B, member 1	Dnajb1	Hspf1, Hsp40	-1.21 (0.143)
NM_008299	DnaJ (Hsp40) homolog, subfamily B, member 3	Dnajb3	Hsj3, Msj1	1.02 (0.519)
NM_025926	DnaJ (Hsp40) homolog, subfamily B, member 4	Dnajb4		1.01 (0.563)
NM_019874	DnaJ (Hsp40) homolog, subfamily B, member 5	Dnajb5	Hsc40, Hsp40-3	1.48 (0.510)
NM_001037940	DnaJ (Hsp40) homolog, subfamily B, member 6	Dnajb6	Mrj, mDj4	-1.01 (0.392)
NM_021317	DnaJ (Hsp40) homolog, subfamily B, member 7	Dnajb7	Dj5, mDj5	1.67 (0.618)
NM_019964	DnaJ (Hsp40) homolog, subfamily B, member 8	Dnajb8	mDj6	-1.13 (0.448)
NM_013760	DnaJ (Hsp40) homolog, subfamily B, member 9	Dnajb9	Mdg1, mDj7, ERdj4	-1.12 (0.287)
NM_020266	DnaJ (Hsp40) homolog, subfamily B, member 10	Dnajb10	Hsj1, mDj8, Dnajb2	-1.19 (0.094)
NM_026400	DnaJ (Hsp40) homolog, subfamily B, member 11	Dnajb11	Dj9, Abbp2	1.07 (0.657)
NM_019965	DnaJ (Hsp40) homolog, subfamily B, member 12	Dnajb12	Dj10, mDj10	1.38 (0.908)
NM_153527	DnaJ (Hsp40) related, subfamily B, member 13	Dnajb13	Tsarg	-1.33 (0.345)
NM_007869	DnaJ (Hsp40) homolog, subfamily C, member 1	Dnajc1	MTJ1, ERdj1, ERj1p, Dnajl1	-1.11 (0.313)
NM_009584	DnaJ (Hsp40) homolog, subfamily C, member 2	Dnajc2	Zrf1, Zrf2, MIDA1	-1.47 (0.471)
NM_008929	DnaJ (Hsp40) homolog, subfamily C, member 3B	Dnajc3a (p58IPK)	p58, mp58, Prkri, p58IPK, Dnajc3, Dnajc3b	-1.09 (0.352)
NM_020566	DnaJ (Hsp40) homolog,	Dnajc4	Hspf2, Mcg18	1.19 (0.707)

	subfamily C, member 4			
NM_016775	DnaJ (Hsp40) homolog, subfamily C, member 5	Dnajc5	Csp	-1.09 (0.340)
NM_025489	DnaJ (Hsp40) homolog, subfamily C, member 5 beta	Dnajc5b		2.00 (0.800)
NM_177677	DnaJ (Hsp40) homolog, subfamily C, member 5 gamma	Dnajc5g		1.12 (0.535)
NM_198412	DnaJ (Hsp40) homolog, subfamily C, member 6	Dnajc6		1.01 (0.601)
NM_019795	DnaJ (Hsp40) homolog, subfamily C, member 7	Dnajc7	Ttc2, mDj11, mTpr2	1.10 (0.716)
NM_172400	DnaJ (Hsp40) homolog, subfamily C, member 8	Dnajc8		-1.05 (0.470)
NM_134081	DnaJ (Hsp40) homolog, subfamily C, member 9	Dnajc9		1.39 (1.000)
NM_024181	DnaJ (Hsp40) homolog, subfamily C, member 10	Dnajc10	JPDI, ERdj5	1.01 (0.483)
NM_172704	DnaJ (Hsp40) homolog, subfamily C, member 11	Dnajc11		1.21 (0.535)
NM_013888	DnaJ (Hsp40) homolog, subfamily C, member 12	Dnajc12	Jdp1, mJDP1	1.00 (0.505)
NM_001163026	DnaJ (Hsp40) homolog, subfamily C, member 13	Dnajc13	Rme8, RME-8, Gm1124	1.41 (0.215)
NM_028873	DnaJ (Hsp40) homolog, subfamily C, member 14	Dnajc14	Dnaj, Hdj3, Lip6, Drip78	1.32 (0.814)
NM_025384	DnaJ (Hsp40) homolog, subfamily C, member 15	Dnajc15	Dnajd1	1.32 (0.997)
NM_172338	DnaJ (Hsp40) homolog, subfamily C, member 16	Dnajc16		1.64 (0.575)
NM_139139	DnaJ (Hsp40) homolog, subfamily C, member 17	Dnajc17		-1.11 (0.418)
NM_029669	DnaJ (Hsp40) homolog, subfamily C, member 18	Dnajc18		1.00 (0.594)
NM_026332	DnaJ (Hsp40) homolog, subfamily C, member 19	Dnajc19	Tim14	1.24 (0.783)
NM_010479	Heat shock protein 1A	Hspa1a	Hsp72, hsp68, Hsp70-3, Hsp70.3, hsp70A1	-1.10 (0.304)
NM_013558	Heat shock protein 1-like	Hspa1l	Msh5, Hsc70t	1.40 (0.670)
NM_008301	Heat shock protein 2	Hspa2	70kDa, Hsp70.2, Hsp70a2, Hsp70-2	-1.02 (0.406)
NM_008300	Heat shock protein 4	Hspa4	70kDa, Apg-2, Hsp110, Hsp70RY	1.09 (0.396)
NM_011020	Heat shock protein 4 like	Hspa4l	94kDa, Apg-1, Osp94	1.19 (0.365)
NM_022310	Heat shock protein 5	Hspa5	Bip, Grp78, Hsce70, Sez7	-1.12 (0.324)
NM_031165	Heat shock protein 8	Hspa8	Hsc70, Hsc71, Hsc73, Hsp73, Hspa10	-1.20 (0.266)
NM_010481	Heat shock protein 9	Hspa9	74kDa, Csa, Grp75, Hsc74, Hsp74, Hsp74a, Hspa9a, Mortalin, Mot2, Nthso70, Pbp74	-1.46 (0.129)
NM_175199	Heat shock protein 12A	Hspa12a	D5Mgi40	1.37 (0.488)
NM_028306	Heat shock protein 12B	Hspa12b		2.05 (0.682)
NM_015765	Heat shock protein 14	Hspa14	70kDa, NST-1, HSP70L1, Hsp70-4	-1.13 (0.401)
NM_013560	Heat shock protein 1	Hspb1	27kDa, Hsp25	-1.14 (0.190)
NM_024441	Heat shock protein 2	Hspb2	Mkbp, 27kDa, Hsp27	2.03 (0.884)
NM_019960	Heat shock protein 3	Hspb3	spb3, Hsbp3	-1.72 (0.258)
NM_001012401	Heat shock protein, alpha-crystallin-related, B6	Hspb6	Gm479, Hsp20	-1.28 (0.053)
NM_013868	Heat shock protein family, member 7 (cardiovascular)	Hspb7	27kDa, cvHsp, Hsp25-2	-1.72 (0.293)
NM_030704	Heat shock protein 8 (Hspb8)	Hspb8	H11, H11K, Cryac, E2ig1, Hsp22, D5ucla4, Hsp20-like	1.35 (0.623)
NM_175111	Hspb associated protein 1	Hspbap1		-1.14 (0.481)
NM_025486	Heat shock protein c171	Hspc171	Tmem208	1.42 (0.992)
NM_010477	Heat shock protein 1 (chaperonin)	Hspd1	60kDa, Hsp60	-1.04 (0.363)

NM_010480	Heat shock protein 90kDa alpha (cytosolic), class A member 1	Hsp90aa1	Hsp4, 86kDa, 89kDa, Hsp89, Hsp90, Hspca, Hsp86-1	-1.10 (0.344)
NM_008302	Heat shock protein 90kDa alpha (cytosolic), class B member 1	Hsp90ab1	90kDa, Hsp84, Hsp90, Hspcb	-1.11 (0.213)
NM_011631	Heat shock protein 90kDa beta (Grp94), member 1	Hsp90b1 (Grp94)	TA-3, Tra1, gp96, ERp99, Grp94, Targ2, Tra-1	-1.22 (0.086)
NM_013559	Heat shock protein 110	Hsp110	105kDa, Hsp105, hsp-E7I, Hsph1	-1.15 (0.186)

Cartilage markers:

NCBI	Gene Name	Gene Symbol	Synonyms	Relative Expression RDW vs WT (PPLR Value)
NM_007424	Aggrecan	Agc1	Agc, cmd, Cspg1, Acan	-1.01 (0.462)
NM_007614	Catenin (cadherin associated protein), beta 1	Ctnnb1	Mesc, Catnb	-1.08 (0.338)
NM_031163	Collagen, type II, alpha 1	Col2a1		-1.23 (0.154)
NM_009933	Collagen, type VI, alpha 1	Col6a1		-1.03 (0.399)
NM_146007	Collagen, type VI, alpha 2	Col6a2		-1.04 (0.414)
XM_897036	Collagen, type VI, alpha 3	Col6a3		-1.17 (0.074)
NM_007739	Collagen, type VIII, alpha 1	Col8a1		-1.06 (0.428)
NM_199473	Collagen, type VIII, alpha 2	Col8a2		1.01 (0.545)
NM_007740	Collagen, type IX, alpha 1	Col9a1		-1.15 (0.232)
NM_007741	Collagen, type IX, alpha 2	Col9a2		1.02 (0.555)
NM_009936	Collagen, type IX, alpha 3	Col9a3		1.00 (0.318)
NM_007729	Collagen, type XI, alpha 1	Col11a1		-1.09 (0.306)
NM_009926	Collagen, type XI, alpha 2	Col11a2		-1.05 (0.354)
NM_016685	Cartilage oligomeric matrix protein	Comp	Tsp5	-1.18 (0.162)
NM_010217	Connective tissue growth factor	Ctgf	Ccn2, Hcs24, Fisp12	-1.01 (0.464)
NM_010056	Distal-less homeobox 5	Dlx5		1.63 (0.901)
NM_008005	Fibroblast growth factor 18	Fgf18		-1.13 (0.335)
NM_008010	Fibroblast growth factor receptor 3	Fgfr3	Hbgfr	-1.19 (0.247)
NM_013500	Hyaluronan and proteoglycan link protein 1	Hapln1	Lp, Clp, Crtl1, Crtl1l	-1.01 (0.518)
NM_010544	Indian hedgehog	Ihh		5.00 (0.930)
NM_010769	Matrilin 1	Matn1	Cmp, Crtm, Mat1	-1.51 (0.000)
NM_016762	Matrilin 2	Matn2	Crtm2	1.71 (0.608)
NM_010770	Matrilin 3	Matn3		-1.22 (0.234)
NM_013592	Matrilin 4	Matn4		1.09 (0.746)
NM_008970	Parathyroid hormone-like peptide	Pthlh	Pthrp	1.21 (0.634)
NM_011199	Parathyroid hormone 1 receptor	Pthr1	Ppr, Pthr, Pth1r	1.34 (0.946)
NM_022017	Transient receptor potential cation channel, subfamily V, member 4	Trpv4	Trp12, Vrl2, Vroac, Otrpc4	-1.08 (0.347)

Hypertrophic cartilage markers:

NM_007431	Alkaline phosphatase, liver/bone/kidney	Akp2	Tnap, Tnsalp, Alpl	1.55 (0.776)
NM_009925	Collagen, type X, alpha 1	Col10a1		-1.61 (0.000)
NM_013599	Matrix metallopeptidase 9	Mmp9	Clg4b	-1.58 (0.146)
NM_008607	Matrix metallopeptidase 13	Mmp13	Clg, Mmp1	-1.35 (0.001)
NM_008608	Matrix metallopeptidase 14	Mmp14	Mt1mmp	1.44 (0.901)
NM_009263	Secreted phosphoprotein 1	Spp1	Op, Bsp, Eta, Opn, Ric, Bnsp, Bspl, Opnl, Apl1	-1.10 (0.337)
NM_009820	Runt related transcription factor 2	Runx2	Cbf, Ls3, Aml3, Osf2, Cbfa1, Pebp2aa, Pebp2a1, Pebpa2a	1.17 (0.516)

Apoptosis:

NCBI	Gene Name	Gene Symbol	Synonyms	Relative Expression RDW vs WT (PPLR Value)
NM_007527	Bcl2-associated X protein	Bax		1.26 (0.926)
NM_009741	B-cell leukemia/lymphoma 2	Bcl2	Bcl-2	-1.21 (0.376)
NM_009808	Caspase 12	Casp12		1.57 (0.707)
NM_009810	Caspase 3	Casp3	Cc3, Lice, Yama, Mldy, Cpp32, Apopain, Caspase-3	1.37 (0.666)
NM_007611	Caspase 7	Casp7	Mch3, Cmh-1, mCasp-7, Ice-lap3, Caspase-7	-1.74 (0.387)
NM_015733	Caspase 9	Casp9	Mch6, Ice-Lap6, Caspase-9	-1.36 (0.563)
NM_007837	DNA damage inducible transcript 3	Ddit3	Chop, Chop10, Chop-10, Gadd153, Ddit3	-1.12 (0.284)
NM_015774	ERO1-like (S. cerevisiae)	Ero1l		1.00 (0.539)
NM_026184	ERO1-like beta (S. cerevisiae)	Ero1lb	Ero1-beta	-1.14 (0.490)
NM_146120	Gelsolin	Gsn		-1.10 (0.201)
NM_010705	Lectin, galactose binding, soluble 3	Lgals3	Gbp, L-34, Gal3, Mac-2	-1.27 (0.123)
NM_008580	Mitogen-activated protein kinase kinase kinase 5	Map3k5	Ask, Ask1, Mekk5, Mapkkk5	-1.59 (0.019)
NM_016700	Mitogen-activated protein kinase 8	Mapk8	Jnk, Jnk1, Prkm8, Sapk1	-1.14 (0.259)
NM_008689	Nuclear factor of kappa light polypeptide gene enhancer in B-cells 1, p105	Nfkb1	p50, p105, Nf-kb1, p50/p105, Nf-kappaB, Nf-kappaB1	-2.01 (0.000)
NM_019408	Nuclear factor of kappa light polypeptide gene enhancer in B-cells 2, p49/p100	Nfkb2	Lyt, p49, p52, p50B, p49/p100, Nf-kappaB2	-1.55 (0.085)
NM_008654	Protein phosphatase 1, regulatory (inhibitor) subunit 15A	Ppp1r15a	Gadd34, Myd116	-1.06 (0.300)
NM_009422	TNF receptor-associated factor 2	Traf2		-1.20 (0.290)
NM_175093	Tribbles homolog 3 (Drosophila)	Trib3	Nipk, Sink, Trb3, Ifld2, Skip3, Trb-3	-1.62 (0.030)
NM_207680	Bcl2-like 11 (apoptosis facilitator)	Bcl2l11	Bim, Bod	-1.59 (0.099)
NM_133234	Bcl2 binding component 3	Bbc3	Puma, Puma/Jfy1	-1.14 (0.363)
NM_026929	Chac, cation transport-like regulator 1 (E.coli)	Chac1		-1.33 (0.022)
NM_019816	Apoptosis antagonizing transcription factor	Aatf	Trb, Che-1	1.09 (0.640)
NM_213659	Signal transducer and activator of transcription 3	Stat3	Aprf	1.69 (0.450)
NM_011716	Wolfram syndrome 1 homolog (human)	Wfs1	Wolframin	1.00 (0.496)
NM_009652	Thymoma viral proto-oncogene 1	Akt1	Akt; PKB; Rac; PKB/Akt; PKBalpha; Akt1	1.00 (0.525)
